# Bithionol eliminates acute myeloid leukaemia stem-like cells by suppressing NF-κB signalling and inducing oxidative stress, leading to apoptosis and ferroptosis

**DOI:** 10.1038/s41420-024-02148-3

**Published:** 2024-08-29

**Authors:** Ingrid R. S. B. Dias, Rafaela G. A. Costa, Ana Carolina B. da C. Rodrigues, Suellen L. R. Silva, Maiara de S. Oliveira, Milena B. P. Soares, Rosane B. Dias, Ludmila F. Valverde, Clarissa A. Gurgel Rocha, Lauren V. Cairns, Ken I. Mills, Daniel P. Bezerra

**Affiliations:** 1 Gonçalo Moniz Institute, Oswaldo Cruz Foundation (IGM-FIOCRUZ/BA), Salvador, Bahia Brazil; 2 SENAI Institute for Innovation in Advanced Health Systems, SENAI CIMATEC, Salvador, BA Brazil; 3 Department of Propaedeutics, Faculty of Dentistry of the Federal University of Bahia (UFBA), Salvador, Bahia Brazil; 4 Department of Biological Sciences, State University of Feira de Santana, Feira de Santana, Bahia Brazil; 5 Department of Dentistry, Federal University of Sergipe, Lagarto, Sergipe Brazil; 6 Center for Biotechnology and Cell Therapy, D’Or Institute for Research and Education (IDOR), Salvador, Bahia Brazil; 7 Patrick G Johnston Centre for Cancer Research, Queen’s University Belfast, Belfast, Northern Ireland UK

**Keywords:** Acute myeloid leukaemia, Cancer stem cells, Pharmacology

## Abstract

Acute myeloid leukaemia (AML) is a lethal bone marrow neoplasm caused by genetic alterations in blood cell progenitors. Leukaemic stem cells (LSCs) are responsible for the development of AML, drug resistance and relapse. Bithionol is an old anthelmintic drug with potential antibacterial, antiviral, antifungal, anti-Alzheimer, and antitumour properties. In this work, we focused on the anti-AML LSC properties of bithionol. This compound inhibited the viability of both solid and haematological cancer cells, suppressed AML stem-like cells, and inhibited AML growth in NSG mice at a dosage of 50 mg/kg, with tolerable systemic toxicity. Bithionol significantly reduced the levels of phospho-NF-κB p65 (Ser529) and phospho-NF-κB p65 (Ser536) and nuclear NF-κB p65 translocation in AML cells, indicating that this molecule can suppress NF-κB signalling. DNA fragmentation, nuclear condensation, cell shrinkage, phosphatidylserine externalisation, loss of transmembrane mitochondrial potential, caspase-3 activation and PARP-(Asp 214) cleavage were detected in bithionol-treated AML cells, indicating the induction of apoptosis. Furthermore, this compound increased mitochondrial superoxide levels, and bithionol-induced cell death was partially prevented by cotreatment with the selective ferroptosis inhibitor ferrostatin-1, indicating the induction of ferroptosis. In addition, bithionol synergised with venetoclax in AML cells, indicating the translational potential of bithionol to enhance the effects of venetoclax in patients with AML. Taken together, these data indicate that bithionol is a potential new anti-AML drug.

## Introduction

Acute myeloid leukaemia (AML) is a bone marrow neoplasm caused by genetic alterations in blood cell progenitors, resulting in overproduction of leukaemic blast cells and impairment of normal haematopoiesis [1–3]. From 2014–2020, this leukaemia had a 5-year survival rate of only 31.9%, with an estimated 20,800 new cases and 11,220 deaths in 2024 in the United States of America [4, 5].

Cytarabine and anthracycline-based chemotherapy have remained the standard treatments for AML for more than 40 years. Since 2017, several targeted therapies, including a BCL-2 inhibitor (venetoclax), FMS-like tyrosine kinase 3 (FLT3) inhibitors (gilteritinib, sorafenib, midostaurin, etc.) and isocitrate dehydrogenase (IDH) inhibitors (IDH1 inhibitor ivosidenib; IDH2 inhibitor enasidenib) [1, 3], have been approved for the treatment of patients with AML. However, due to the high heterogeneity of this disease, new drugs are urgently needed.

Leukaemic stem cells (LSCs) are responsible for the development of AML, uncontrolled proliferation, drug resistance and relapse. In particular, the high recurrence rate in patients with AML is because current therapies are LSC sparing, and even the small number of LSCs that survive to initial chemotherapy spread rapidly or remain dormant after treatment [6–8].

Interestingly, NF-κB signalling is constitutively active in AML LSCs but not in normal haematopoietic progenitor cells, indicating that the NF-κB pathway might be used as a selective target for anti-AML LSCs to kill this subset population [6, 9, 10]. In addition, AML LSCs exhibit low oxidative stress levels and are susceptible to drugs that cause oxidative stress. As a result, cellular redox homoeostasis has been identified as a key potential target for eliminating AML LSCs [11].

**Fig. 1:**
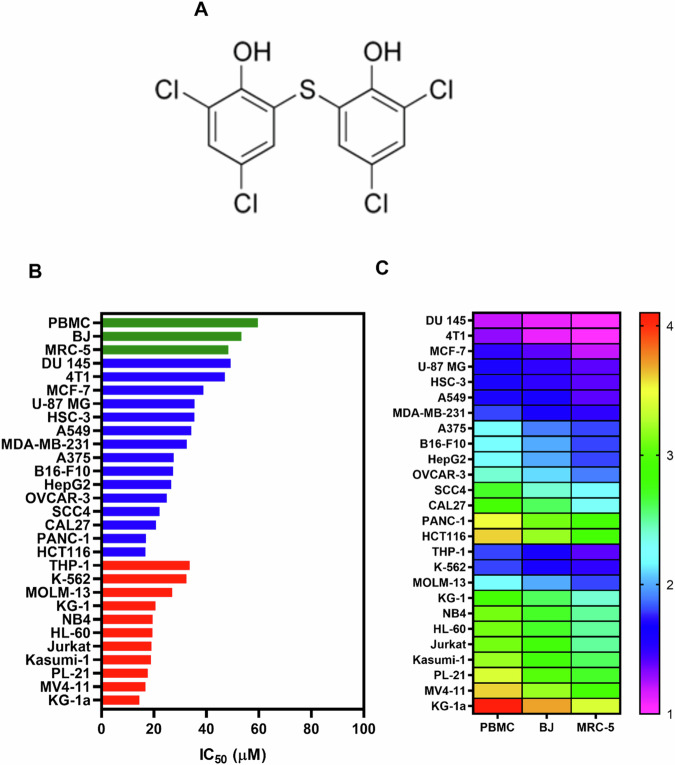
Bithionol is cytotoxic to solid and haematological cancer cells. **A** Chemical structure of bithionol. **B** IC_50_ values for the cytotoxicity of bithionol against haematological (red bars) and solid cancer cells (blue bars), as well as against three different populations of noncancerous cells (green bars). **C** Heatmap of selectivity indices calculated for bithionol.

Bithionol (Fig. 1A) is an old anthelmintic drug that, despite reports of its use in horses [12, 13], is no longer used in humans due to efficiency issues [14]. It has also been used in cosmetics as an antimicrobial agent but was removed because it caused photosensitivity when used topically [15]. In addition, the potential antibacterial [16], antiviral [17], antifungal [18], anti-Alzheimer [19], and antitumour [20, 21] properties of bithionol have been reported in preclinical studies.

Bithionol displayed cytotoxicity to ovarian cancer cells, synergising with cisplatin and paclitaxel by inhibiting NF-κB signalling and inducing oxidative stress [20, 22, 23]. Furthermore, bithionol was also shown to be cytotoxic to cervical cancer cell lines through the inhibition of NF-κB signalling [24]. Therefore, as the inhibition of NF-κB signalling [10] and the induction of oxidative stress [11] are targets for AML LSC elimination, we hypothesised that bithionol could suppress the AML LSC subpopulation. In this study, we focused on the anti-AML LSC properties of bithionol.

## Results

### Bithionol is cytotoxic to solid and haematological cancer cells

The cytotoxicity of bithionol was assessed in a panel of 17 solid cancer cell lines (MCF-7, MDA-MB-231, 4T1, HCT116, B16-F10, A375, HepG2, OVCAR-3, U-87 MG, A549, PANC-1, DU145, HSC-3, CAL27, SCC4, SSC-9, and SSC-25), 11 haematological cancer cell lines (Jurkat, MOLM-13, KG-1, KG-1a, Kasumi-1, NB4, HL-60, K-562, THP-1, PL-21, and MV4-11), and three different populations of noncancerous cells (PBMC, BJ, and MRC-5) after 72 h of incubation, including both human and mouse cell lines (Fig. 1B and Table S1). In solid cancer cells, bithionol exhibited half-maximal inhibitory concentration (IC_50_) values ranging from 16.7 to >70 µM for human colorectal carcinoma HCT116 cells and human oral squamous cell carcinoma SCC9/SCC25 cells, respectively. In haematological malignant cells, bithionol had IC_50_ values ranging from 14.4 to 33.6 µM for human acute myelogenous leukaemia KG-1a cells and human monocytic leukaemia THP-1 cells, respectively. Doxorubicin, which was used as a positive control, had IC_50_ values ranging from 0.01 to 3.5 μM for human acute myelogenous leukaemia MOLM-13 cells and human acute myeloblastic leukaemia Kasumi-1 cells, respectively.

In noncancerous cells, bithionol presented IC_50_ values of 59.6, 53.4 and 48.4 μM for human PBMCs, human foreskin fibroblast BJ cells and human lung fibroblast MRC-5 cells, respectively. Doxorubicin had IC_50_ values of 1, 1.6 and 1.3 μM for PBMCs, BJ cells and MRC-5 cells, respectively. The selectivity indices (SIs) were calculated and are shown in Fig. 1C and Table S2. The SI of bithionol was greater than twofold for most malignant cells, indicating good selectivity.

### Bithionol reduces the number of AML stem-like cells and suppresses AML growth in NSG mice

To evaluate the effect of bithionol on AML progenitor/stem cells, the effect of bithionol on KG-1a cells was explored, as this AML cell line expresses a CD34+ stem-like phenotype [25]. First, the cytotoxic effects of bithionol at concentrations of 7, 14 and 28 μM were confirmed in KG-1a cells by trypan blue exclusion assay after 12, 24, 48 and 72 h of incubation (Fig. S1). Bithionol reduced the number of viable cells in a time- and concentration-dependent manner. After 12 h of treatment, the viability inhibition was 20.2, 22.4, and 23.3%, respectively, whereas it was 20.8, 27.8, and 39.4%, respectively, after 24 h of treatment. After 48 h of treatment, the viability inhibition was 37.5, 56.5 and 71.3%, whereas it was 79.9, 81.9 and 84.2% after 72 h of treatment, respectively.

**Fig. 2:**
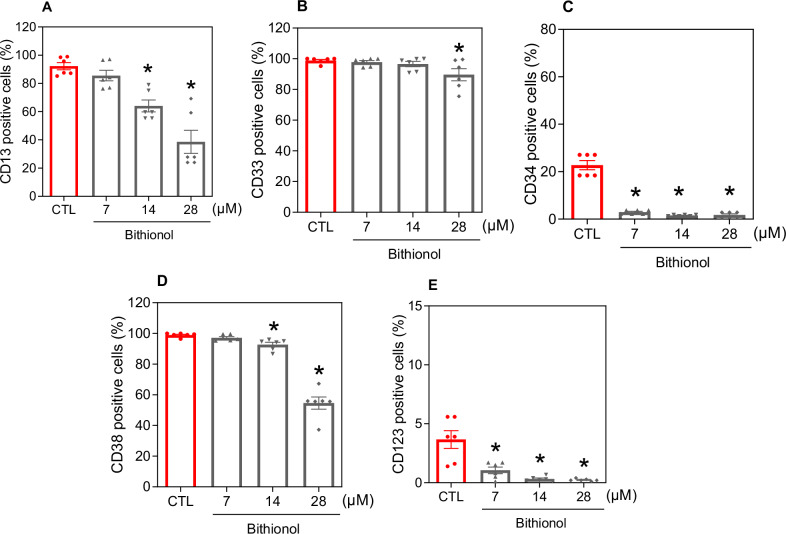
Bithionol reduces the number of AML stem-like cells. Immunophenotypic analysis of the myeloid lineage markers CD13 (**A**) and CD33 (**B**) and the AML progenitor/stem markers CD34 (**C**), CD38 (**D**) and CD123 (**E**) in bithionol-treated KG-1a cells after 48 h of incubation. The vehicle (0.2% DMSO) was used as a negative control (CTL). The data are shown as the mean ± SEM of three biological replicates carried out in duplicate. *p < 0.05 compared with CTL by one-way ANOVA followed by Dunnett’s multiple comparisons test.

Next, flow cytometric immunophenotyping was performed in bithionol-treated KG-1a cells after 48 h of incubation with the myeloid lineage markers CD13 [26] and CD33 [27] and the AML progenitor/stem markers CD34 [28], CD38 [28], and CD123 [29] (Fig. 2). CD34 and CD123 are LSC-specific markers, whereas CD38 identifies proliferative progenitor cells but not LSCs. Importantly, bithionol decreased the proportion of KG-1a cells positive for CD34 and CD123, indicating the suppression of AML progenitor/stem cells.

To verify whether bithionol was capable of inducing cell differentiation, the expression of the maturation marker CD11b [30] was quantified in KG-1a cells treated with bithionol for 24 h (Fig. S2). However, bithionol did not affect the expression of CD11b, indicating that its effect is not related to the induction of the differentiation of myeloid progenitors.

**Fig. 3:**
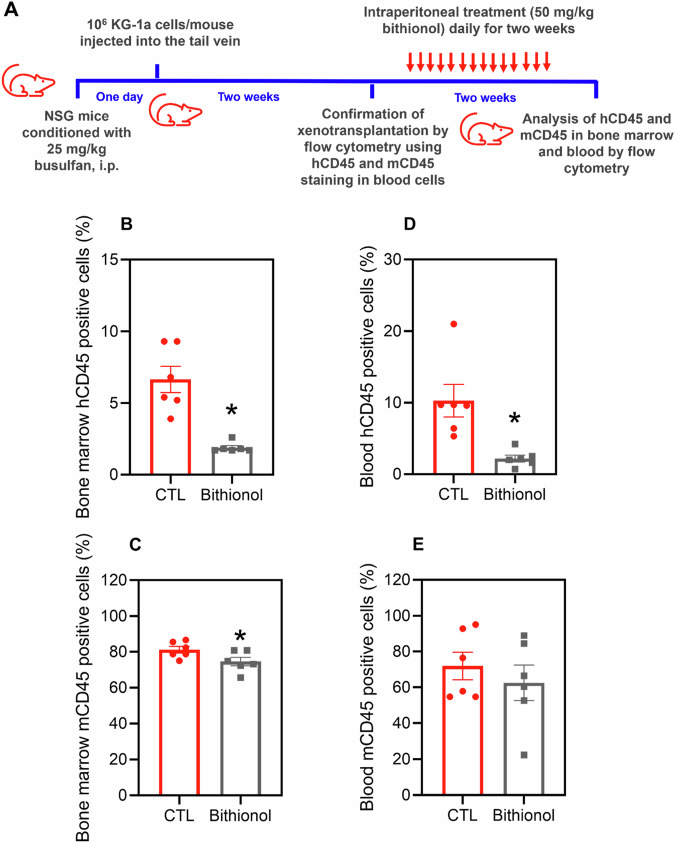
Effect of bithionol on the growth of xenografts derived from KG-1a cells. **A** A xenograft model was established in NSG mice. Two weeks after the inoculation of KG-1a cells, the mice were randomly divided into a bithionol (50 mg/kg) group and a control group (5% DMSO). The treatments were injected into the mice intraperitoneally every day for two weeks. hCD45-positive cells in the bone marrow (**B**) and peripheral blood (**D**) and mCD45-positive bone marrow (**C**) and peripheral blood (**E**) cells were quantified by flow cytometry. The data are shown as the mean ± SEM of 6 animals. *p < 0.05 compared with CTL by Student’s *t* test.

The in vivo anti-AML effects of bithionol were assessed in a leukaemia xenograft model using NSG mice engrafted with KG-1a cells, and anti-human CD45 (hCD45) and anti-mouse CD45 (mCD45) antibodies were used to quantify human leukaemic blasts and mouse leucocytes, respectively (Fig. 3). Curiously, treatment with 50 mg/kg bithionol for two weeks significantly reduced the percentage of hCD45+ cells in the bone marrow and peripheral blood of NSG mice. These data indicate the ability of bithionol to eliminate leukaemic blasts in mice. All animals in the control and bithionol-treated groups showed 100% survival.

With respect to toxicological aspects, no significant change in body weight or organ weight was detected, except in the kidneys (Figure S3). Furthermore, histopathological analysis of the kidneys, lungs, and liver of bithionol-treated and control animals revealed similar alterations, the majority of which were minor and/or reversible, indicating little damage to normal tissues (Fig. S4). No histological changes were observed in the hearts of any of the animals (data not shown).

### Bithionol suppresses NF-κB signalling in AML cells

**Fig. 4:**
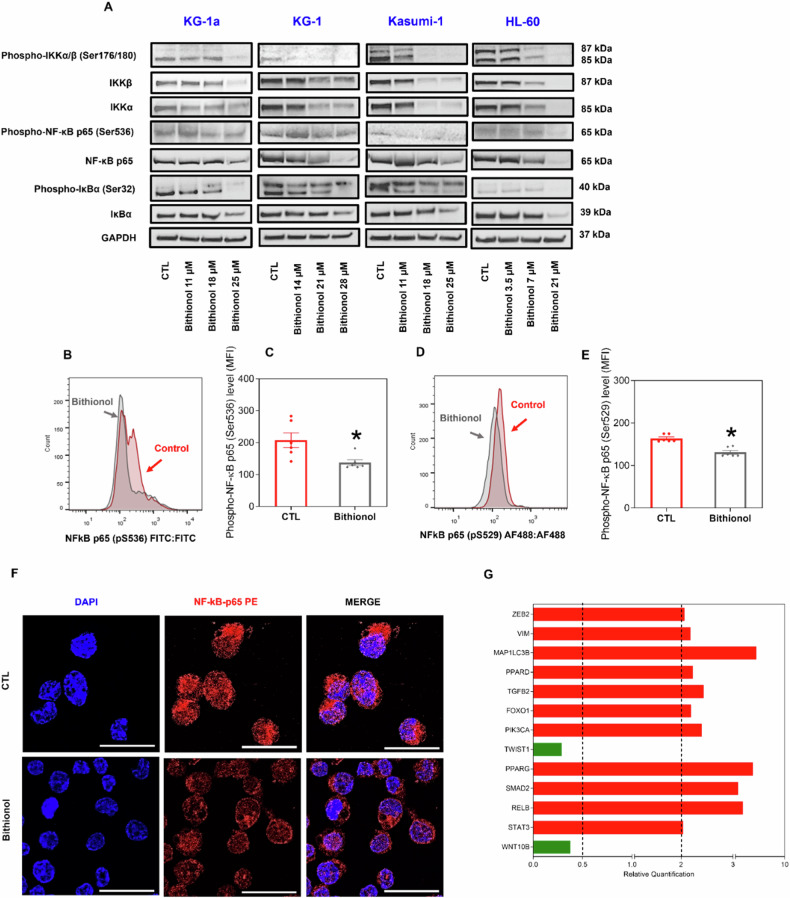
Bithionol inhibits NF-κB signalling in AML cells. **A** Western blot of phospho-IKKα/β (Ser176/180), IKKα, IKKβ, NF-κB p65, phospho-NF-κB p65 (Ser536), phospho-IκBα (Ser32) and IκBα in the AML KG-1a, KG-1, Kasumi-1 and HL-60 cell lines treated with bithionol for 72 h. GAPDH was used as an internal control. **B**, **C** Effect of bithionol (28 μM) on the levels of phospho-NF-κB p65 (Ser536) after 24 h of treatment in KG-1a cells, as assessed by flow cytometry. **D**, **E** Effect of bithionol (28 μM) on the levels of phospho-NF-κB p65 (Ser529) after 24 h of treatment in KG-1a cells, as assessed by flow cytometry. The vehicle (0.2% DMSO) was used as a negative control (CTL). The data are shown as the mean ± SEM of three biological replicates carried out in duplicate. * *p* < 0.05 compared with CTL by Student’s *t* test. MFI = mean fluorescence intensity. **F** Representative immunofluorescence images of NF-κB p65 in KG-1a cells after 24 h of incubation with 28 μM bithionol. Scale bar = 25 μm. **G** Up- and downregulated genes in KG-1a cells after 12 h of treatment with 28 µM bithionol. Genes that displayed RQ ≥ 2 (red bars) were upregulated, and those that displayed RQ ≤ 0.5 (green bars) were downregulated.

As bithionol has been reported to be an inhibitor of NF-κB [20, 24], we investigated whether this molecule could inhibit this signalling pathway in AML cells. For this purpose, we evaluated the protein expression levels of IKKα, IKKβ, IκBα and NF-κB p65, as well as the levels of IKKα/β phosphorylated at Ser 176/180; IκBα phosphorylated at Ser 32; and NF-κB p65 phosphorylated at Ser 536 in AML KG-1a, KG-1, Kasumi-1, and HL-60 cells by western blot after 72 h of bithionol treatment (Fig. 4A). Furthermore, after 24 h of bithionol treatment, the levels of NF-κB p65 phosphorylated at Ser536 (Fig. 4B, C) and NF-κB p65 phosphorylated at Ser529 (Fig. 4D, E) were quantified in KG-1a cells via flow cytometry, and the nuclear translocation of NF-κB p65 (Figs. 4F and S5) was investigated in KG-1a cells via confocal microscopy. Both NF-κB p65 phosphorylation and nuclear translocation suggest the activation of NF-κB p65 cell signalling [31].

Bithionol reduced the protein expression of elements of the NF-κB p65 signalling pathway, as well as the phosphorylation of key elements of this pathway, causing a decrease in the nuclear translocation of NF-κB p65, indicating that bithionol can suppress NF-κB p65 signalling in AML cells.

The molecular mechanism of bithionol in KG-1a cells was also investigated via a qPCR array using a panel of 92 genes related to cell signalling, epithelial–mesenchymal transition and/or cell death pathways selected on the basis of data from AML LSC gene expression analysis. A total of 11 upregulated genes and two downregulated genes were found in KG-1a cells after treatment with 28 μM bithionol for 12 h (Fig. 4G and Table S3). Although the expression of the *RELB* gene (RQ = 4.05) increased, no significant changes were found in the expression of the *NFKB1* (RQ = 1.56), *NFKB2* (RQ = 1.66), *NFKBIA* (RQ = 1.82), *NFKBIB* (RQ = 0.96), or *RELA* (RQ = 1.31) genes, suggesting that bithionol does not affect the NF-κB signalling pathway at the transcriptional level. *WNT10B* (RQ = 0.38) and *TWIST1* (RQ = 0.29) were among the bithionol-downregulated genes, suggesting that these compounds could interfere with Wnt signalling and cell motility, respectively.

### Bithionol induces caspase-mediated apoptosis in AML cells

**Fig. 5:**
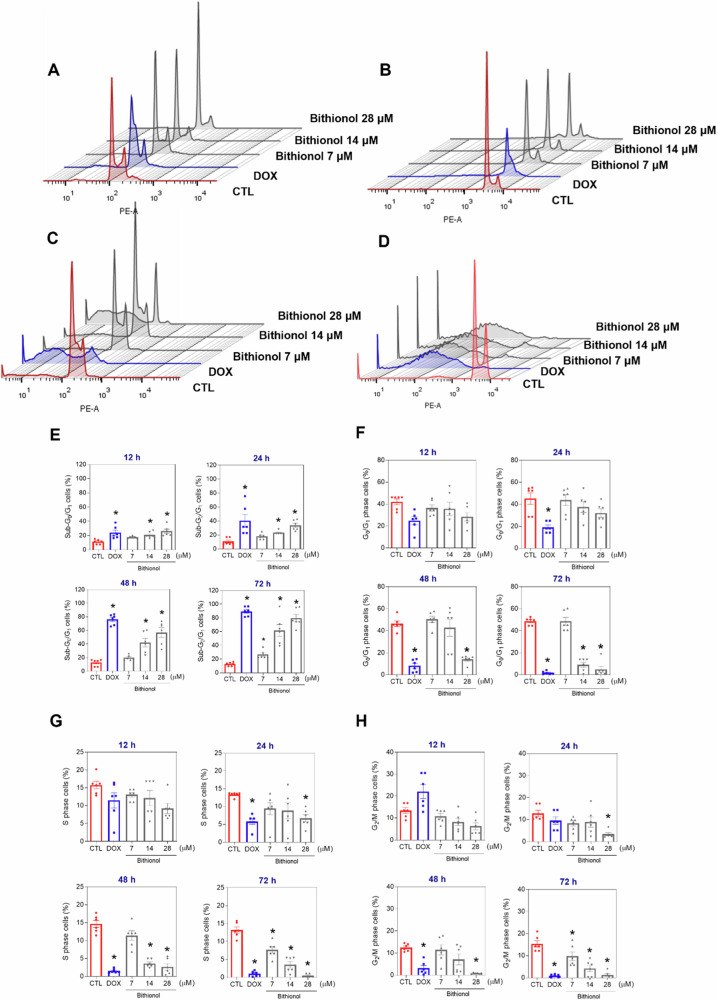
Cell cycle progression in KG-1a cells after treatment with bithionol. Representative histograms after 12 (**A**), 24 (**B**), 48 (**C**), and 72 (**D**) h of treatment. Percentages of cells in sub-G_0_/G_1_ (**E**), G_0_/G_1_ (**F**), S (**G**), and G_2_/M (**H**) after different incubation periods with bithionol. Vehicle (0.2% DMSO) was used as a negative control (CTL), and doxorubicin (DOX, 1 µM) was used as a positive control. The data are shown as the mean ± S.E.M. of three biological replicates carried out in duplicate. *p < 0.05 compared with CTL by one-way ANOVA followed by Dunnett’s multiple comparisons test.

The distribution of DNA content was analysed in KG1a cells after 12, 24, 48, and 72 h of treatment by flow cytometry to determine the cell cycle phases, and all hypodiploid cells (<2n) were considered cells with fragmented DNA (sub-G_0_/G_1_). Significant concentration- and time-related induction of DNA fragmentation was observed with all concentrations of bithionol used (Fig. 5A‒H). At concentrations of 7, 14 and 28 μM, bithionol caused DNA fragmentation by 17.8%, 20.9%, and 26.1%, respectively, after 12 h of incubation (in contrast to the 11.2% found in the control); by 18.1%, 23.1%, and 33.7%, respectively, after 24 h of incubation (in comparison to the 10.5% found in the control); by 20.1%, 42.2%, and 57.0%, respectively, after 48 h of incubation (in comparison to the 12.8% detected in the control); and by 28.6%, 62.0%, and 79.7%, respectively, after 72 h of incubation (in comparison to the 12.8% detected in the control). Consequently, cell cycle phases (G_0_/G_1_, S and G_2_/M) decreased proportionally in KG-1a cells treated with bithionol. Doxorubicin, which was used as a positive control, also caused DNA fragmentation in KG-1a cells.

Cell size and cellular granularity/complexity were measured via flow cytometry forward scattering (FSC) and side scattering (SSC), respectively, in KG1-a cells after 12, 24, 48, and 72 h of treatment with bithionol. The morphological changes induced by bithionol in KG-1a cells included nuclear condensation, as indicated by the increase in SSC, and cell shrinkage, as indicated by the reduction in FSC (Fig. S6). These morphological features are compatible with apoptotic cell death.

**Fig. 6:**
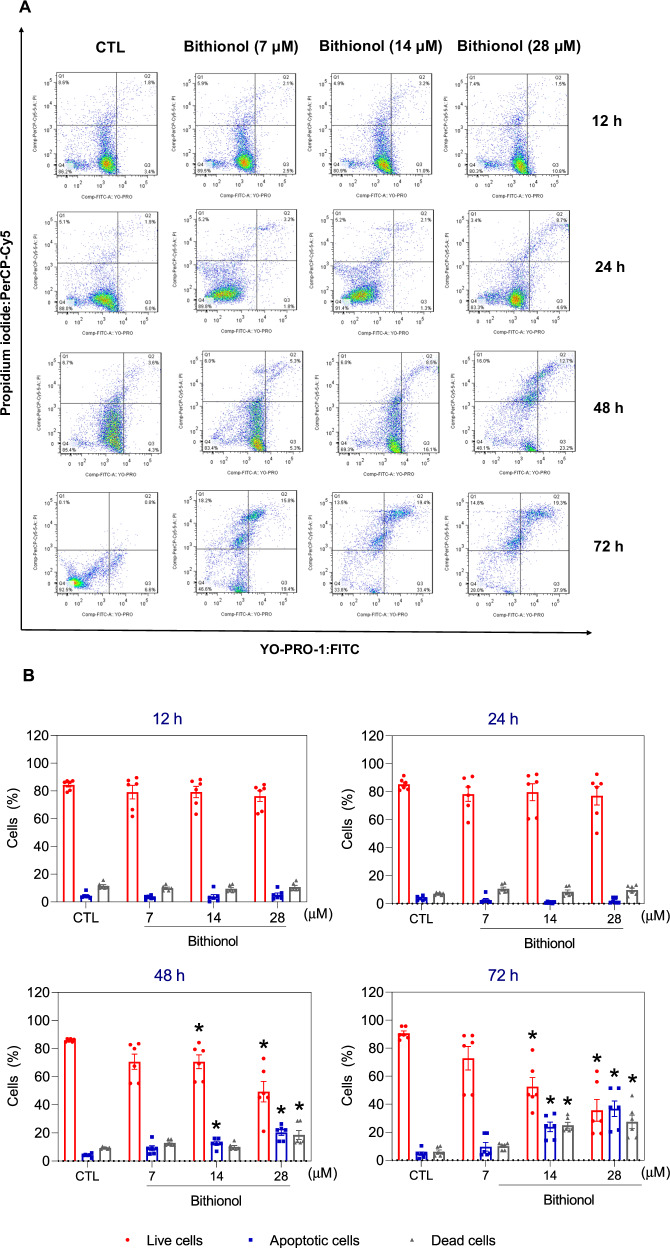
Bithionol induced apoptotic cell death in KG-1a cells. **A** Representative flow cytometry dot plots. **B** Apoptosis quantification in KG-1a cells after 12, 24, 48, and 72 h of treatment with bithionol. Quantification of live (YO-PRO-1/PI double negative cells), apoptotic (YO-PRO-1-positive/PI-negative cells), and dead (YO-PRO-1/PI double positive cells plus PI-positive/YO-PRO-1 negative cells) KG-1a cells. Vehicle (0.2% DMSO) was used as a negative control (CTL). The data are shown as the mean ± SEM of three biological replicates carried out in duplicate. *p < 0.05 compared with CTL by one-way ANOVA followed by Dunnett’s multiple comparisons test.

Furthermore, YO-PRO-1/propidium iodide (PI) double staining was used to investigate apoptotic cell death [32] in bithionol-treated KG-1a cells after 12, 24, 48 and 72 h of incubation. Live (YO-PRO-1/PI double negative cells), apoptotic (YO-PRO-1-positive/PI-negative cells), and dead (YO-PRO-1/PI double positive cells plus PI-positive/YO-PRO-1 negative cells) cells were quantified. Bithionol significantly increased the percentage of apoptotic cells after 48 and 72 h of incubation (no significant changes were observed after 12 and 24 h of incubation) (Fig. 6A, B). At concentrations of 7, 14, and 28 μM, bithionol caused apoptosis by 9.1%, 12.7%, and 20.3%, respectively, after 48 h of incubation (in contrast to the 4.1% found in the control), whereas 9.6%, 23.9%, and 36.8%, respectively, were found after 72 h of incubation (in contrast to the 4.7% found in the control). In addition, bithionol induced phosphatidylserine externalisation in AML KG-1a, KG-1, Kasumi-1, and HL-60 cells after 72 h of incubation (Fig. S7).

**Fig. 7:**
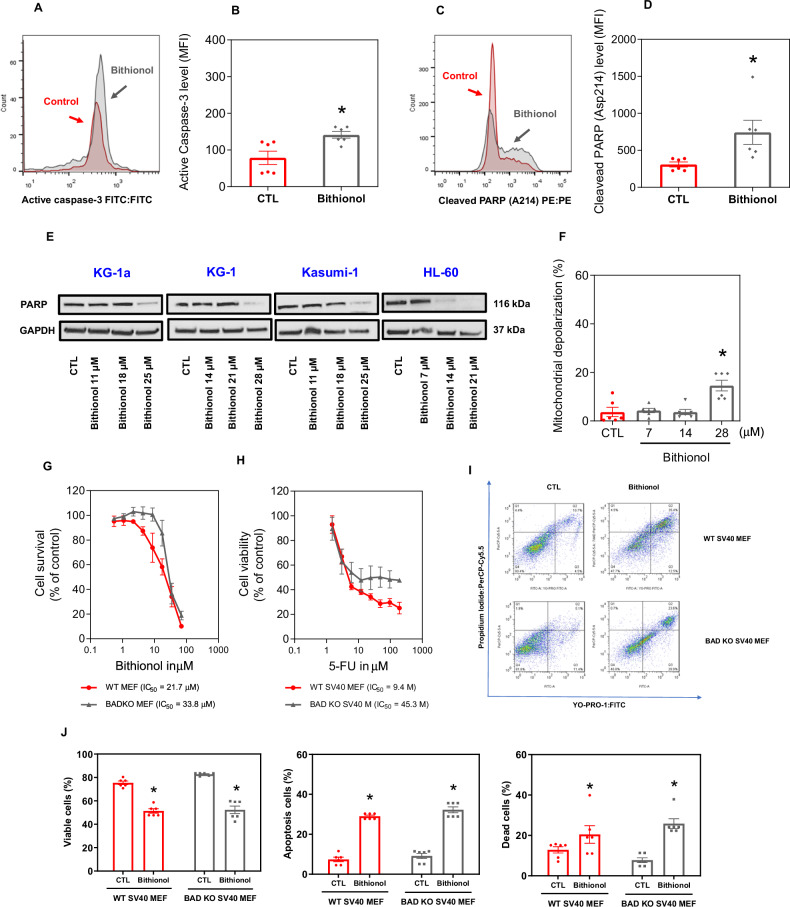
Bithionol causes caspase-mediated apoptosis in AML cells. **A**, **B** Effects of bithionol (28 μM) on the levels of active caspase 3 and (**C**, **D**) cleaved PARP (Asp214) after 24 h of treatment in KG-1a cells, as assessed by flow cytometry. *p < 0.05 compared with CTL by Student’s *t* test. MFI = mean fluorescence intensity. **E** Western blot of PARP from AML KG-1a, KG-1, Kasumi-1 and HL-60 cells treated with bithionol for 72 h. GAPDH was used as an internal control. **F** Effect of bithionol on mitochondrial activity in KG-1a cells after 24 h of incubation. *p < 0.05 com*p*ared with CTL by one-way ANOVA followed by Dunnett’s multiple comparisons test. **G**, **H** Concentration‒response curves of WT SV40 MEFs and BAD KO SV40 MEFs upon treatment with 5-fluorouracil (5-FU, a positive control) and bithionol. The curves were obtained from at least three biological replicates carried out in duplicate via the Alamar blue assay after 72 h of incubation. **I**, **J** Induction of cell death in the WT SV40 MEFs and BAD KO SV40 MEFs after 48 h of incubation with 40 μM 5-FU and 28 μM bithionol. *p < 0.05 compared with CTL by Student’s *t-*test. Vehicle (0.2% DMSO) was used as a negative control (CTL). The data are shown as the mean ± SEM of three biological replicates carried out in duplicate.

The levels of active caspase-3 and cleaved PARP (Asp 214), which are markers of apoptosis induction [33], were investigated via flow cytometry in bithionol-treated KG-1a cells. Increased active caspase-3 (Fig. 7A, B) and cleaved PARP-(Asp 214) (Fig. 7C, D) were detected in KG-1a cells treated with bithionol for 24 h. Furthermore, a reduction in PARP was detected by western blot in KG-1a, KG-1, Kasumi-1, and HL-60 cells treated with bithionol for 72 h (Fig. 7E). Similarly, caspase-3 was measured by western blot in KG-1a cells treated with bithionol for 72 h and was also reduced (Fig. S8).

Next, the pancaspase inhibitor Z-VAD(OMe)-FMK was used to evaluate the role of caspases in bithionol-induced apoptotic cell death in KG-1a cells (Fig. S9). Although no changes in the percentage of viable cells were detected, cotreatment with Z-VAD(OMe)-FMK decreased the percentage of cells undergoing apoptosis caused by bithionol and increased the percentage of dead cells.

As mitochondrial dysfunction plays a central role in the induction of apoptosis [34], we quantified the transmembrane mitochondrial potential of KG-1a cells treated with bithionol for 24 h. A significant reduction in the transmembrane mitochondrial potential was found in bithionol-treated KG-1a cells (Fig. 7F), confirming that this molecule can cause apoptotic cell death.

Furthermore, members of the BCL-2 family of proteins are essential regulators of pro- and antiapoptotic activity [35] and have also been investigated. Although bithionol treatment did not significantly affect the transcriptional levels of BCL-2 family members (Table S3), the BCL-2 protein level was reduced in bithionol-treated KG-1a cells after 72 h of treatment (Fig. S8). As mouse embryonic fibroblasts are effective models for exploring gene knockout functions, the role of the proapoptotic protein BAD was investigated in a BAD gene knockout immortalised mouse embryonic fibroblast line (BAD-KO SV40 MEFs) in comparison to its parental wild-type immortalised mouse embryonic fibroblast line (WT SV40 MEFs). On the other hand, bithionol-induced cell death seemed to be BAD independent since this molecule exhibited similar cytotoxicity to that in BAD KO SV40 MEFs and to that in parental control WT SV40 MEFs (Fig. 7G‒J).

### Bithionol induces ferroptosis in AML cells

**Fig. 8:**
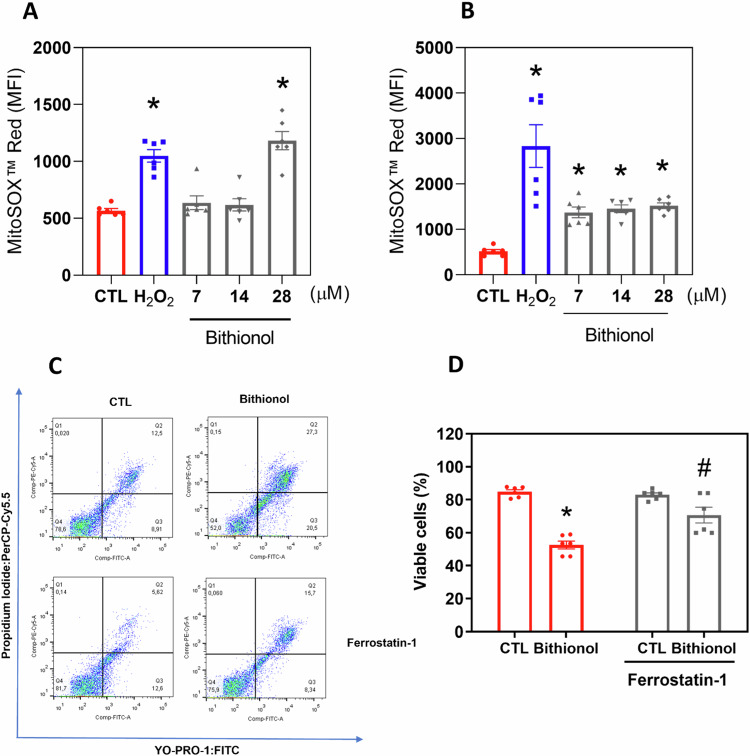
Bithionol causes ferroptosis in AML cells. Mitochondrial ROS in KG-1a cells after 1 (**A**) and 24 (**B**) h of treatment with bithionol. **C**, **D** Effects of the selective ferroptosis inhibitor ferrostatin-1 on the death of KG-1a cells induced by bithionol. The cells were pretreated for 2 h with 1 μM ferrostatin-1 and then co-incubated with 28 μM bithionol for 72 h. Vehicle (0.2% DMSO) was used as a negative control (CTL), and hydrogen peroxide (H_2_O_2_, 100 µM) was used as a positive control. The data are shown as the mean ± SEM of three biological replicates carried out in duplicate. *p < 0.05 compared with CTL by one-way ANOVA followed by Dunnett’s multiple comparisons test. ^#^*p* < 0.05 com*p*ared with the respective treatment without inhibitor by one-way ANOVA followed by Dunnett’s multiple comparisons test. MFI mean fluorescence intensity.

As bithionol is a pro-oxidant agent [20, 22, 23], we investigated its effect on the redox activity of AML KG-1a cells. First, mitochondrial superoxide levels in bithionol-treated KG-1a cells were quantified by MitoSOX^TM^ Red after 1 and 24 h of incubation. Importantly, bithionol increased mitochondrial superoxide levels in KG-1a cells. At concentrations of 7, 14 and 28 μM, the MFIs of bithionol-treated KG-1a cells were 636.8, 617.7, and 1183, respectively (in contrast to the 566.3 detected in the control), after 1 h of incubation (Fig. 8A), whereas 1374, 1453, and 1521, respectively (in contrast to the 517.3 detected in the control), were detected after 24 h of incubation (Fig. 8B), indicating the induction of oxidative stress by bithionol in AML cells.

Next, oxidative stress-mediated ferroptosis was investigated in KG-1a cells subjected to bithionol-induced cytotoxicity via the selective ferroptosis inhibitor ferrostatin-1 (a lipid peroxidation inhibitor) [36]. In particular, the coincubation of bithionol with ferrostatin-1 partially prevented bithionol-induced cell death, indicating that this compound can induce ferroptosis-related cell death in AML cells (Fig. 8C, D).

### Bithionol synergises with venetoclax in AML cells

**Fig. 9:**
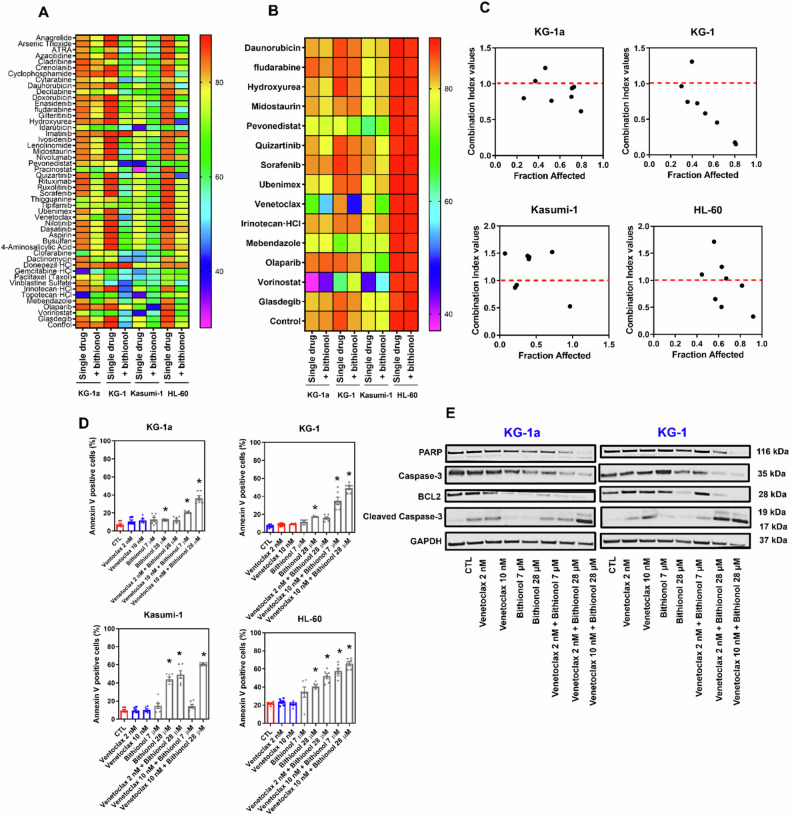
Bithionol synergises with venetoclax in AML cells. **A** Heatmap of the results of the drug combination assay carried out in KG-1a, KG-1, Kasumi-1, and HL-60 cells after 72 h of incubation with bithionol (28 μM) and 48 selected drugs (2 nM) of different pharmacological classes, the majority of which are already used to treat myeloid malignancies. **B** Heatmap of the results of the drug combination assay carried out in KG-1a, KG-1, Kasumi-1, and HL-60 cells after 72 h of incubation with bithionol (28 μM) and 14 selected drugs (2 nM). **C** Combination index plot of the interaction between venetoclax and bithionol in KG-1a, KG-1, Kasumi-1, and HL-60 cells after 72 h of incubation. The synergistic, additive, and antagonistic effects of drugs are defined by combination index values of <1.0, 1.0, and >1.0, respectively. **D** Phosphatidylserine externalisation induced by bithionol combined with venetoclax in KG-1a, KG-1, Kasumi-1, and HL-60 cells after 72 h of incubation. The data are shown as the mean ± SEM of three biological replicates carried out in duplicate. *p < 0.05 compared with CTL by one-way ANOVA followed by Dunnett’s multiple comparisons test. **E** Western blot of the PARP, caspase-3, cleaved caspase-3, and BCL-2 proteins in KG-1a and KG-1 cells after treatment with bithionol combined with venetoclax. GAPDH was used as an internal control.

As drug combination studies are critical translational strategies for eradicating neoplastic cells, drug combination assays were carried out in AML KG-1a, KG-1, Kasumi-1, and HL-60 cells after 72 h of incubation with bithionol (28 μM) and 48 drugs (2 nM) of different pharmacological classes, the majority of which are already used to treat myeloid malignancies. The combination of quizartinib, daunorubicin, olaparib, midostaurin, mebendazole, fludarabine, pevonedistat, vorinostat, hydroxyurea, sorafenib, ubenimex, venetoclax, irinotecan, and glasdegib with bithionol significantly reduced cell viability (Fig. 9A and Table S4). These drugs were selected and tested in an additional experiment (Fig. 9B and Table S5), where venetoclax was the best combination option.

Venetoclax was selected for calculating the combination index in AML KG-1a, KG-1, Kasumi-1, and HL-60 cells after 72 h of incubation. Curiously, the combination of bithionol (7, 14, and 28 µM) with venetoclax (2, 10, and 50 nM) had synergistic effects on most of the concentration combinations tested (Fig. 9C and Table S6). Furthermore, an increase in phosphatidylserine externalisation (Fig. 9D) and a decrease in PARP, caspase-3, and BCL-2 proteins, as well as an increase in cleaved caspase-3 (Fig. 9E), were detected in the group treated with venetoclax combined with bithionol.

## Discussion

Herein, we report that bithionol is cytotoxic to solid and haematologic cancer cells and can reduce the number of AML stem-like cells and suppress AML growth in NSG mice. Bithionol suppressed NF-κB signalling and caused apoptosis and ferroptosis in AML cells. Furthermore, bithionol synergised with venetoclax in AML cells, indicating that this compound has important anti-AML effects.

Bithionol was previously reported to be a cytotoxic agent against cisplatin-resistant (OVACAR-3, SKOV-3, A2780-CDDP and IGROV-1CDDP) or cisplatin-sensitive (A2780 and IGROV-1) ovarian cancer cells [20], as well as against the cervical cancer cell lines ME180 and HeLa [24]. Moreover, this compound induced apoptosis in SKOV-3 ovarian cancer cells engrafted into Foxn1^nu^ nude mice, although it was not able to inhibit tumour growth or improve mouse survival at dosages of 30–240 mg/kg [21]. In this study, we reported that bithionol could inhibit the viability of both solid and haematological cancer cells and, for the first time, that bithionol could suppress AML stem-like cells and inhibit AML growth in NSG mice at a dosage of 50 mg/kg with tolerable systemic toxicity. Indeed, although contact photosensitisation is observed when bithionol is administered topically [15], people who receive multiple oral doses of bithionol experience low toxicity and no skin irritation [37].

Mechanistically, Ayyagari et al. [20] demonstrated that bithionol induced ovarian cancer cell death by increasing active caspase 3/7 and PARP cleavage; leading to nuclear condensation; the loss of mitochondrial potential; cell cycle arrest at the G_0_/G_1_ phase; and the upregulation of p38, p27, and p21 proteins, as well as the downregulation of phospho-Akt, phospho-NF-κB, phospho-IκBα, XIAP, BCL-2 and BCL-XL. Increased oxidative stress and autotaxin inhibition were also found in bithionol-treated ovarian cancer cells [20]. In addition, photoactivation of bithionol increased oxidative stress and cytotoxicity in keratinocytes [38–40].

Furthermore, Miller et al. [24] reported that bithionol depolarised the mitochondrial membrane potential, increased caspase 3/7 activity in cervical cancer cells, and suppressed NF-κB signalling through the inhibition of IκBα phosphorylation, as detected by cell-based reporter gene assays. Indeed, we found that bithionol did not affect the transcription of NF-κB signalling elements but reduced the phosphorylation level of key elements of this pathway, including IκBα. Therefore, these data corroborate that bithionol alters NF-κB signalling by inhibiting the phosphorylation of IκBα.

In our study, we found that bithionol suppresses NF-κB signalling and oxidative stress induction, causing apoptosis and ferroptosis in AML cells. Both NF-κB cell signalling inhibition and the induction of oxidative stress are targets for eliminating AML LSCs [6, 9–11], confirming the ability of bithionol to reduce the number of AML stem-like cells.

Interestingly, increased activation of NF-κB signalling by some cytotoxic chemotherapies has been reported as a resistance mechanism; therefore, some NF-κB inhibitors can overcome this chemoresistance phenotype [41–43]. Bithionol synergises with cisplatin and paclitaxel in ovarian cancer cells by inhibiting NF-κB signalling and inducing oxidative stress [22, 23]. Here, we observed that bithionol synergises with venetoclax in AML cells. Importantly, a mechanism of venotoclax resistance is also related to increased activation of NF-κB cell signalling [44–46]. These findings indicate the translational potential of bithionol in enhancing the effects of venetoclax in patients with AML.

These data indicate that bithionol is a potential new anti-AML drug targeting NF-κB inhibition and oxidative stress induction that can reduce the number of AML stem-like cells and synergise with venotoclax.

## Materials and methods

### Bithionol

Bithionol was obtained commercially (Sigma‒Aldrich Co., Saint Louis, MO, USA).

### Cells

Table S7 details all the cells used in this study, including 11 haematological cancer cell lines, 17 solid cancer cell lines, three different populations of noncancerous cells and one mutant cell line and its parental control. All the cell lines were cultured according to ATCC animal cell culture guidelines and were maintained at 37 °C in a 5% CO_2_ atmosphere. To maintain exponential growth, the cells were replicated every 3‒4 days, and 0.25% trypsin EDTA solution (Sigma‒Aldrich Co.) was used to detach adherent cells. Mycoplasma contamination was monitored by a mycoplasma staining kit (Sigma Aldrich Co.), and only cell lines that tested negative for mycoplasma were used.

### Cell viability assay

Cell viability was quantified via CellTiter-Glo luminescent, Alamar blue or trypan blue exclusion assays. For the CellTiter-Glo® luminescent assay (Promega, Southampton, UK), the cells were analysed following the manufacturer’s instructions. For the Alamar blue assay, the cells were added to 96-well culture plates (7 × 10^3^ cells/well for adherent cells or 3 × 10^4^ cells/well for nonadherent cells) and maintained at 37 °C in a 5% CO_2_ atmosphere. Bithionol was added to each well at eight different concentrations and incubated for 72 h. Doxorubicin (purity ≥ 95%, Laboratory IMA S.A.I.C., Buenos Aires, Argentina) was used as a positive control. Four hours before the end of the incubation period (or 24 h for PBMCs), resazurin (Sigma‒Aldrich Co.) was added to each well at a final concentration of 3 μM. The absorbance values at 570 nm and 600 nm were evaluated via a SpectraMax 190 Microplate Reader (Molecular Devices, Sunnyvale, CA, USA).

For the trypan blue exclusion assay, 90 μL of the cell suspension was added to 10 μL of trypan blue (0.4%), and viable (unstained cells) and nonviable (trypan blue-stained cells) cells were counted in a haemocytometer via light microscopy.

### Flow cytometry assays

A panel of fluorochrome-conjugated primary monoclonal antibodies (Table S8) against CD13, CD33, CD34, CD38, and CD123 was used to immunophenotype the KG-1a cells. Figure S10 presents the gate strategy used. CD11b expression was also analysed via additional staining. For cell staining, the cells were washed with PBS containing 0.5% bovine serum albumin and incubated with antibodies for 1 h at room temperature. The cells were subsequently washed with PBS, stained with YO-PRO-1 (Sigma‒Aldrich Co.) and analysed via flow cytometry. A BD LSRFortessa or BD LSR II flow cytometer with BD FACSDiva Software (BD Biosciences, San Jose, CA, USA) and FlowJo Software 10 (FlowJo LCC, Ashland, OR, USA) were used. Single cells were selected from H-FSC versus A-FSC or H-SSC versus A-SSC. Cellular debris was also excluded from the analyses. At least 3 × 10^4^ events were acquired/sample.

For intracellularly stained cells, fluorochrome-conjugated primary monoclonal antibodies (Table S8) were also used. Briefly, the cells were centrifuged and resuspended in 0.5–1 mL of 4% formaldehyde for 10 min at 37 °C. After washing, the tube was placed on ice for 1 min. The cells were permeabilized on ice for 30 min by gently vortexing ice-cold 100% methanol into precooled cells until the final methanol concentration reached 90%. After washing with PBS containing 0.5% bovine serum albumin, primary antibodies were added, and the samples were incubated for 1 h at room temperature. Next, the cells were washed with PBS and analysed by flow cytometry, as detailed above. At least 10^4^ events were acquired per sample.

Internucleosomal DNA fragmentation and cell cycle phases were evaluated by flow cytometry via PI staining [47]. A solution containing 0.1% Triton X-100, 2 µg/mL PI, 0.1% sodium citrate, and 100 µg/mL RNase (all from Sigma Aldrich Co.) was used to stain the cells. After 15 min of incubation in the dark, the cells were analysed via flow cytometry as described above. At least 10^4^ events were acquired per sample.

Cell viability was evaluated by an annexin V-FITC/PI (FITC Annexin V Apoptosis Detection Kit I, BD Biosciences) or YO-PRO-1/PI (Sigma‒Aldrich Co.) double staining following the manufacturer’s instructions. The cells were analysed by flow cytometry, as detailed above. At least 10^4^ events were acquired per sample. For functional assays, the selective ferroptosis inhibitor ferrostatin-1 (Sigma‒Aldrich Co.) and the pancaspase inhibitor Z-VAD(OMe)-FMK (Cayman Chemical; Ann Arbor, MI, USA) were used.

The mitochondrial transmembrane potential was investigated in rhodamine 123-stained cells [48]. Briefly, cells stained with 1 μg/mL rhodamine (Sigma‒Aldrich Co.) for 15 min at 37 °C in the dark were washed and analysed by flow cytometry, as detailed above. At least 10^4^ events were acquired per sample.

Mitochondrial superoxide levels were evaluated by MitoSOX™ Red reagent (Thermo Fisher Scientific, Waltham, MA, USA) following the manufacturer’s instructions. The cells were analysed by flow cytometry, as detailed above. At least 10^4^ events were acquired per sample.

### AML xenograft model

Twelve specific pathogen-free NOD. Cg-Prkdc^scid^ Il2rg^tm1Wjl^/SzJ (NSG) mice (male and female, 20–25 g) were acquired and kept at FIOCRUZ-BA animal facilities (Salvador, Bahia, Brazil), and a local animal ethics committee approved the experimental protocol (#16/2018). All the animals were fed a standard pellet diet with food and water available ad libitum. An artificially lit room (12 h dark/light cycle) was used.

All animals were treated with 25 mg/kg busulfan (Sigma Aldrich Co.) one day before receiving KG-1a cells to allow for high bone marrow engraftment [49]. The next day, the mice were inoculated with 10^6^ cells/mouse through the tail vein. Every day, all the animals were checked for signs of weight loss or lethargy. Flow cytometry was used to confirm engraftment in peripheral blood after two weeks via both PE-conjugated hCD45 and FITC-conjugated mCD45 monoclonal antibodies (Table S8), as described above. At least 3 × 10^4^ events were acquired per sample.

After engraftment confirmation, the animals were randomly divided into two groups (n = 6): group 1 was given vehicle (5% DMSO, a negative control), and group 2 was given bithionol at a dosage of 50 mg/kg. Every day for two weeks, the animals received intraperitoneal treatments. The mice were subsequently euthanised with an anaesthetic overdose (thiopental, 100 mg/kg), and cells from the peripheral blood and bone marrow were collected. These cells were stained with hCD45 and mCD45 antibodies and evaluated by flow cytometry, as detailed above. At least 3 × 10^4^ events were acquired per sample.

Furthermore, kidneys, lungs, hearts, and livers were collected for toxicological investigation. These organs were examined for colour change, gross lesion formation, and/or bleeding, fixed in 4% formaldehyde, dehydrated in a graded alcohol series, washed in xylene, and coated in paraffin wax. Tissues were cut into 5 μm thick slices, stained with haematoxylin‒eosin and/or periodic acid‒Schiff (liver and kidney), and histologically evaluated under a light microscope.

### Western blot

The cells were lysed for protein extraction with 60 µL of RIPA buffer (Thermo Fisher Scientific). The protein concentration was determined via a BCA protein assay kit (Merck, Poole, UK). Each sample was subjected to 4‒12% polyacrylamide gel electrophoresis (SDS‒PAGE) (Life Technologies, Renfrewshire, UK), transferred to a nitrocellulose membrane (Hybond-C, Amersham; UK) and incubated with primary antibodies followed by secondary antibodies (Table S9). Protein bands were assessed via a Pierce ECL detection kit (Thermo Scientific; Leicestershire, UK). The membranes were scanned via a benchtop G:box (Syngene, Cambridge, UK).

### Immunofluorescence by confocal microscopy

Immunofluorescence was evaluated to analyse the localisation of NF-κB p65. The cells were washed twice with PBS before being plated as droplets (5 μL) on coverslips, permeabilized with Triton X-100 (0.5%), treated with RNase (10 μg/mL), washed with PBS, and incubated overnight with an anti-NF-κB p65 antibody (Table S8). The cells were washed with PBS the next day and mounted with Fluoromount-G (Invitrogen, Thermo Fisher Scientific) containing DAPI. A Leica TCS SP8 confocal microscope (Leica Microsystems, Wetzlar, HE, Germany) was used to photograph the cells.

### qPCR array

Total RNA was isolated via the RNeasy Plus Mini Kit (Qiagen; Hilden, Germany) following the manufacturer’s instructions. A NanoDrop® 1000 spectrophotometer (Thermo Fisher Scientific, Waltham, MA, USA) was used to evaluate and quantify RNA purity. A Superscript VILO^TM^ kit (Invitrogen Corporation; Waltham, MA, USA) was used for RNA reverse transcription.

Gene expression was evaluated via qPCR analysis via a TaqMan® Array Plate 96 plus fast (#4413256, Applied Biosystems™, Foster City, CA, USA) on an ABI ViiA7 system (Applied Biosystems™). The PCR cycling conditions were 50 °C for 2 min and 95 °C for 10 min, followed by 40 cycles of 95 °C for 15 s and 60 °C for 1 min. The 2^−ΔΔCT^ method [50] with Gene Expression Suite™ software (Applied Biosystems™) was used to calculate the relative quantification (RQ) of mRNA expression. The calibrators were cells treated with 0.2% DMSO (negative control). For data normalisation, the geometric mean RQs of the three reference genes *GUSB*, *HPRT1*, and *GAPDH* were used. All the experiments were carried out under DNase/RNase-free conditions. It was determined that genes would be upregulated if the RQ was ≥2, meaning that gene expression in drug-treated cells was at least twofold greater than that in negative control-treated cells. Similarly, genes were determined to be downregulated when RQ < 0.5, meaning that gene expression in drug-treated cells was at least half that of negative control cells.

### Drug combination assay

The drug combination assay was performed in 384-well black optical bottom plates (Nunc, Science Warehouse Limited). The cells were seeded at a density of 2 × 10^5^ cells/well. A combination of 48 drugs (2 nM) of different pharmacological classes, most already used to treat myeloid malignancies, with bithionol (28 µM) was tested. The drugs were diluted with DMSO (Merck, Poole, UK) and added to the cells to generate the desired compound concentration. Cells treated with 0.1% DMSO were used as vehicle controls. All drug treatments were performed via an ECHO liquid handling robot (Labcyte). After the drugs were combined with bithionol, the cells were incubated for 72 h, and cell cytotoxicity was assessed via the CellTox™ Green Cytotoxicity Assay (Promega, Southampton, UK) according to the manufacturer’s instructions. The relative fluorescence unit (RFU) (Ex: 485 nm, Em: 520 nm) was measured via a Synergy HTX Multi-Mode Micro-Plate reader (Biotek, VT, USA).

To calculate the combination index (CI), the cells were plated at a density of 3 × 10^5^ cells/well and treated with venetoclax (2, 10 or 50 nM), bithionol (7, 14 or 28 µM) or combinations thereof. The cells were then incubated for 72 h, after which cell viability was assessed via the CellTiter-Glo luminescent assay, as described above.

For potential synergistic or additive effects, we used CompuSyn software (version 1.0; ComboSyn, Paramus, NJ, USA). Isobolograms and CI plots were generated, and CI values were calculated via the Chou-Talalay method [51]. CI values < 1 indicate synergy, CI values = 1 indicate an additive effect, and CI values > 1 indicate an antagonistic effect.

### Statistical analysis

The results are shown as the mean ± SEM or as IC_50_ values with a 95% confidence interval from at least three independent experiments (biological replicates) carried out in duplicate (technical replicates). For statistical analyses, a two-tailed unpaired Student’s *t* test (for data with two groups) or one-way ANOVA followed by Dunnett’s multiple comparisons test (for data with three or more groups) was applied via GraphPad Prism (Intuitive Software for Science; San Diego, CA, USA). The SIs were calculated via the following formula: SI = IC_50_ [noncancerous cells]/IC_50_ [cancer cells]. For gene expression analysis, a gene was upregulated if its RQ was ≥2. Similarly, genes were downregulated when RQ ≤ 0.5.

### Supplementary information

Original WB

SUPPLEMENTAL MATERIAL

## Data Availability

The data will be made available from the corresponding author upon reasonable request.
