# Supplementary material for: Bithionol eliminates acute myeloid leukaemia stem-like cells by suppressing NF-κB signalling and inducing oxidative stress, leading to apoptosis and ferroptosis
Source: Cell Death Discov. 2024 Aug 29;10:390. doi: 10.1038/s41420-024-02148-3 (PMC11362533; doi:10.1038/s41420-024-02148-3)

Figure 4A KG-1a cells

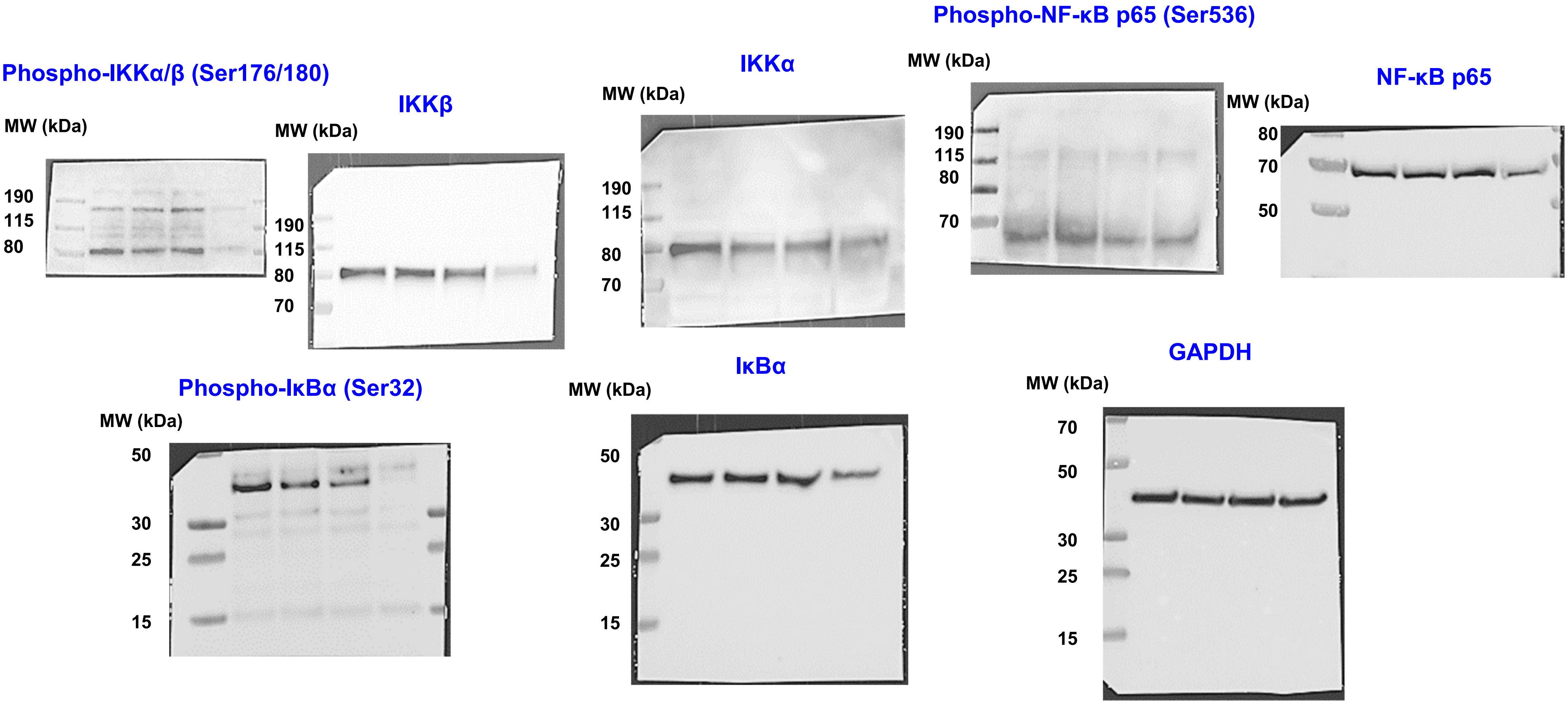

Figure 4A KG-1 cells

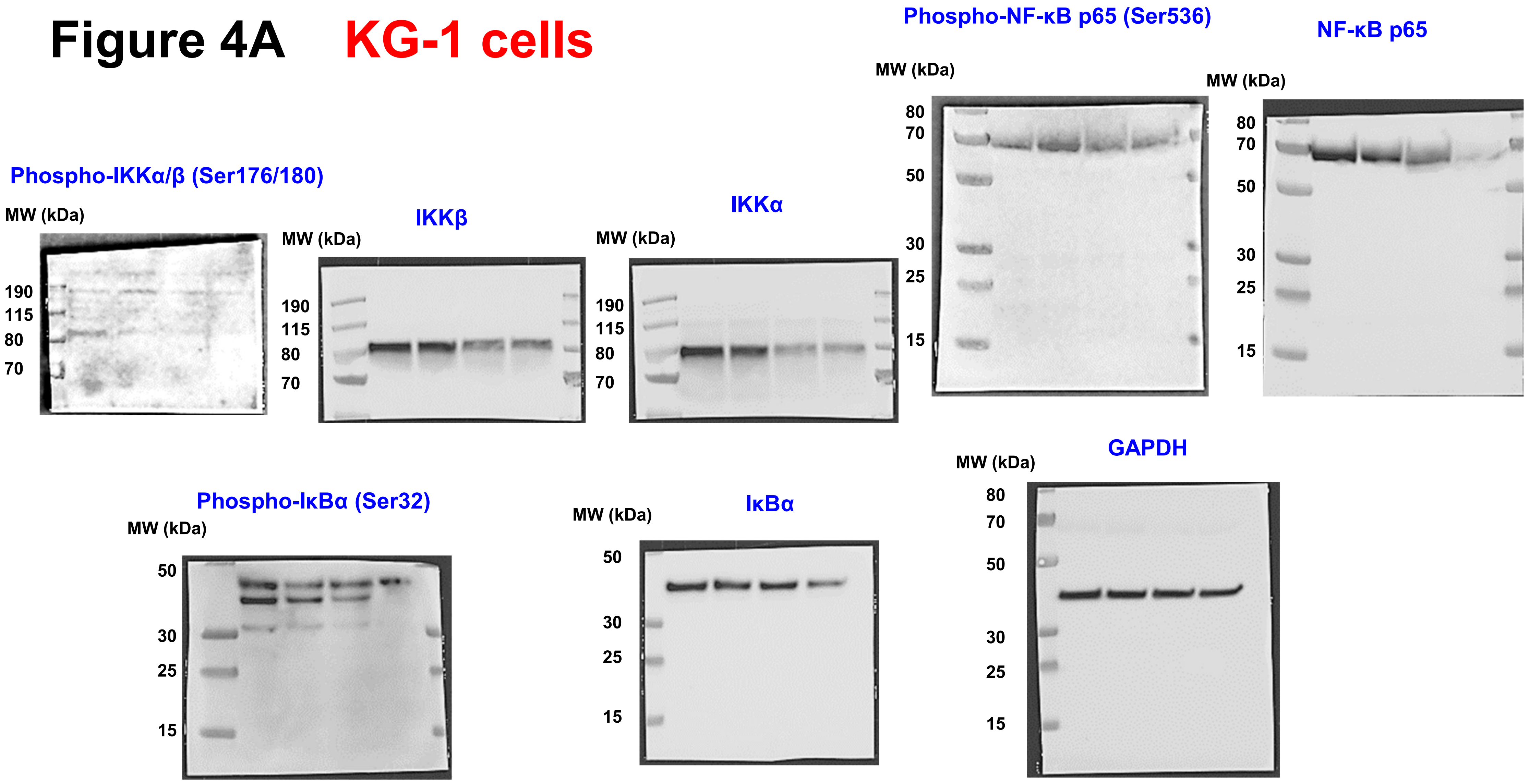

Figure 4A Kasumi-1 cells

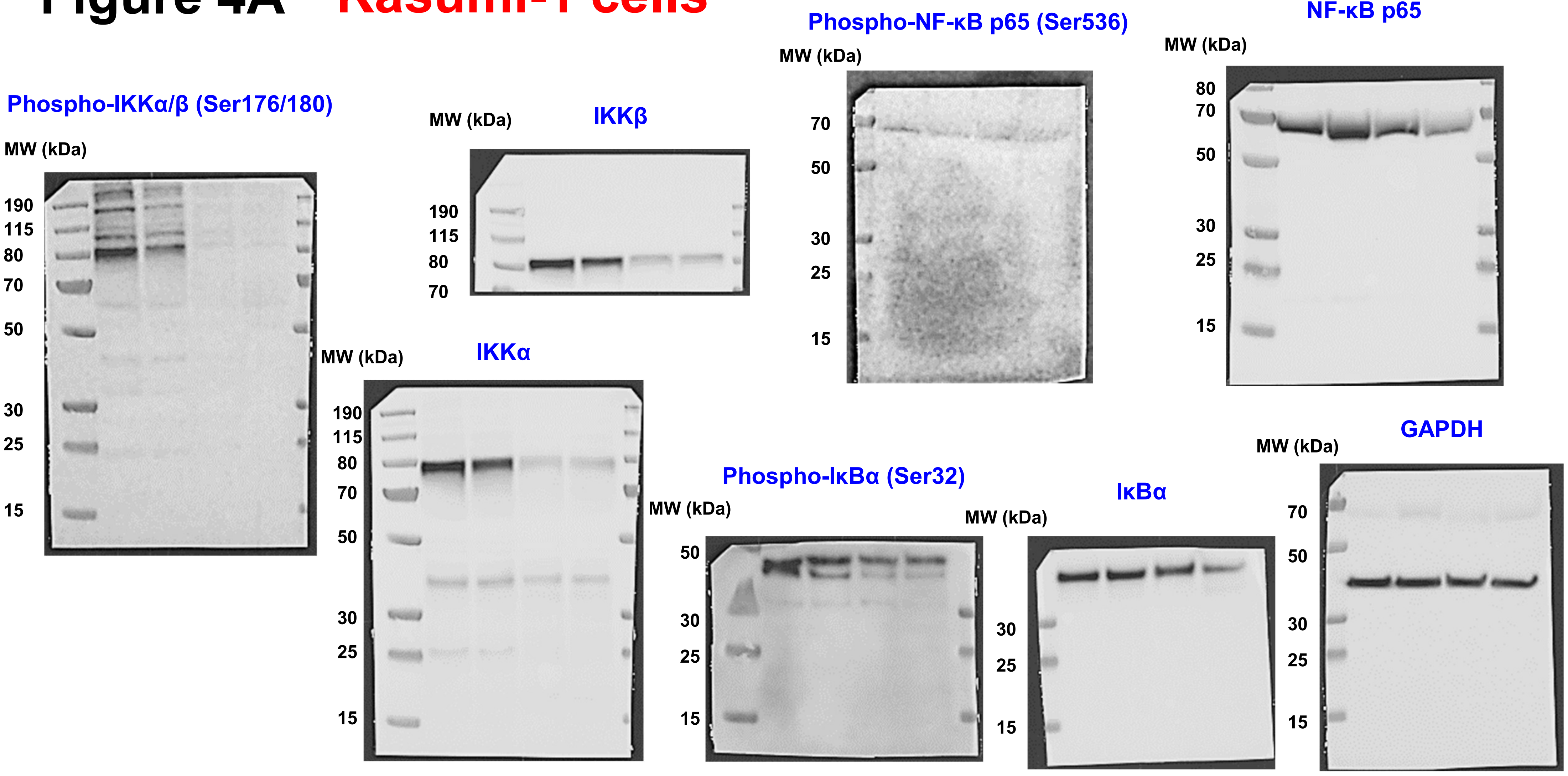

Figure 4A HL-60 cells

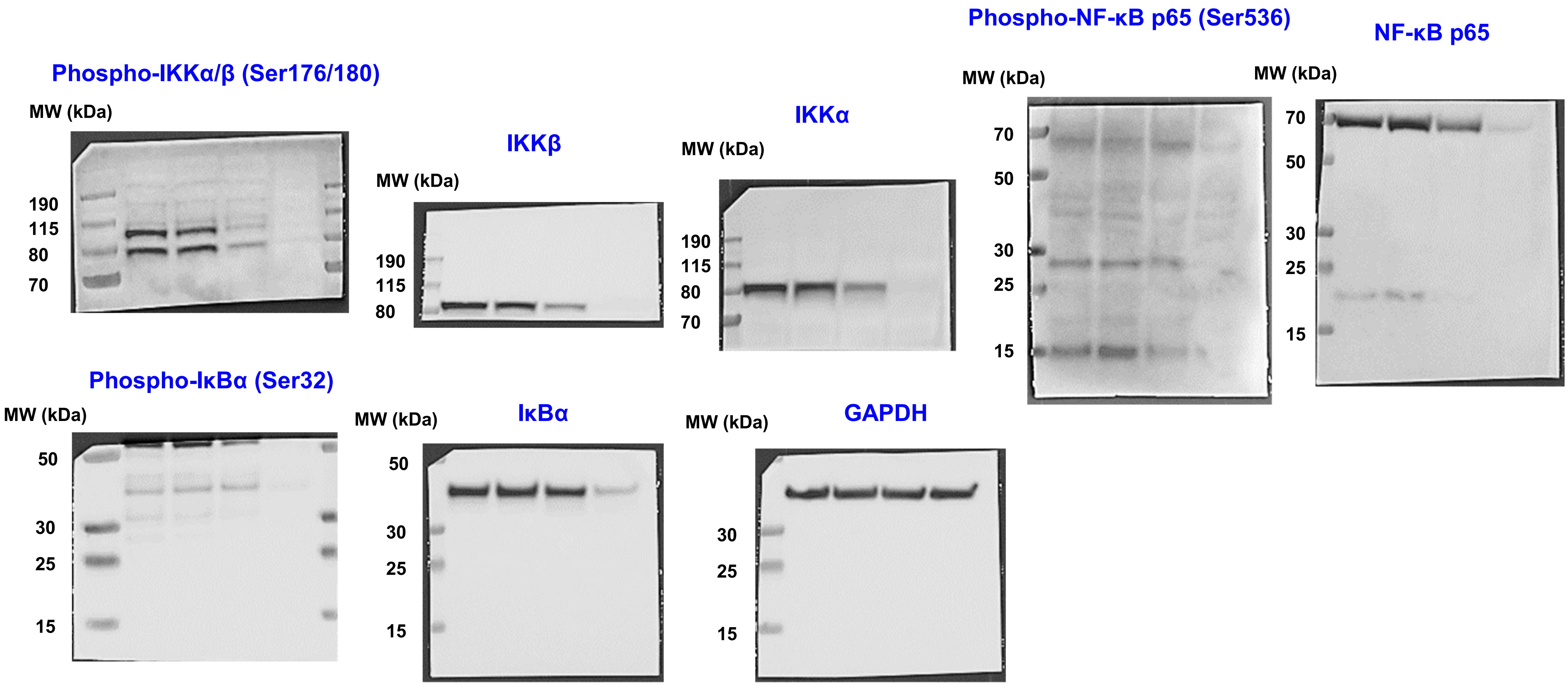

Figure 7E

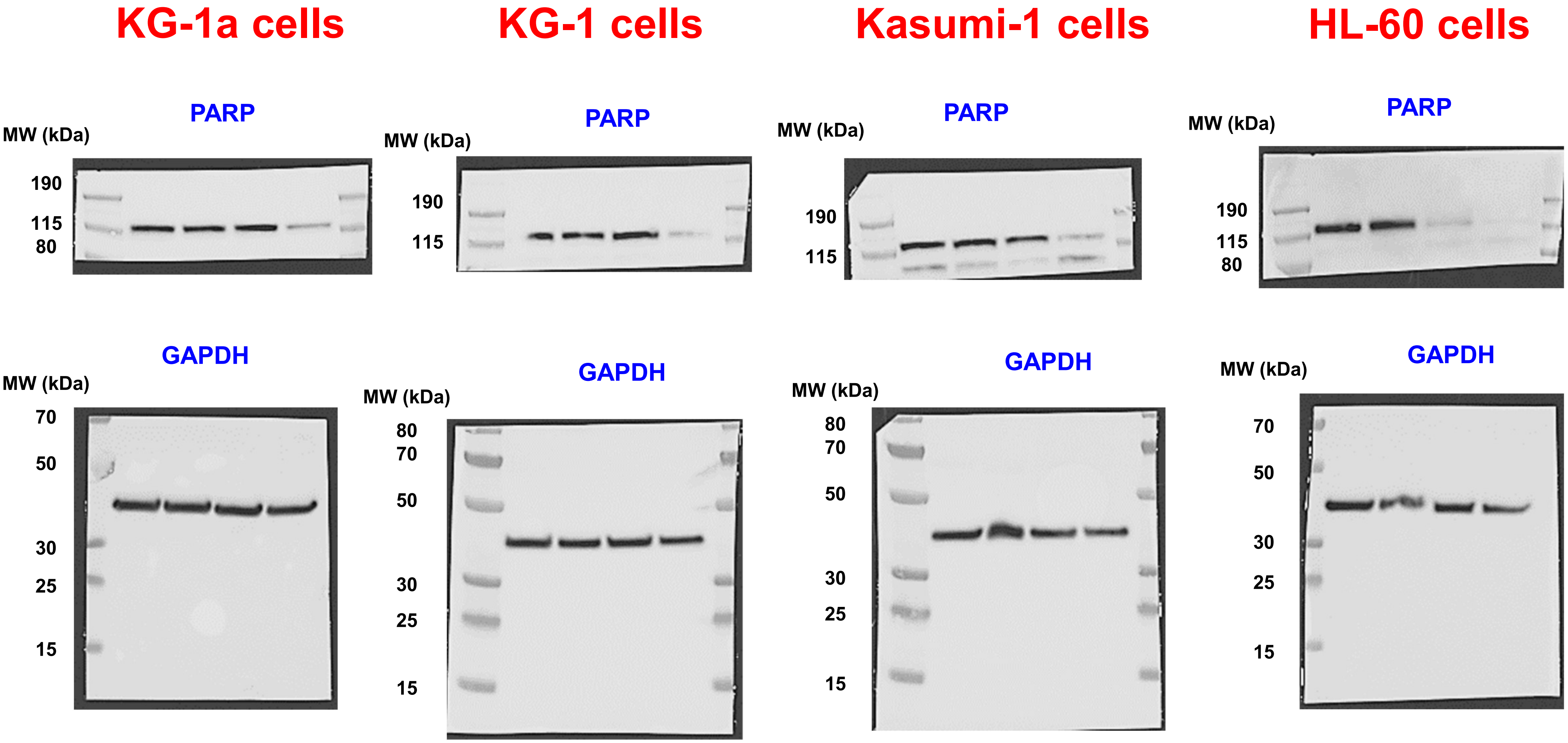
KG-1a cells
KG-1 cells
Kasumi-1 cells
HL-60 cells
PARP
MW (kDa)
190
115
80
GAPDH
70
50
30
25
15

Figure 9E KG-1a cells

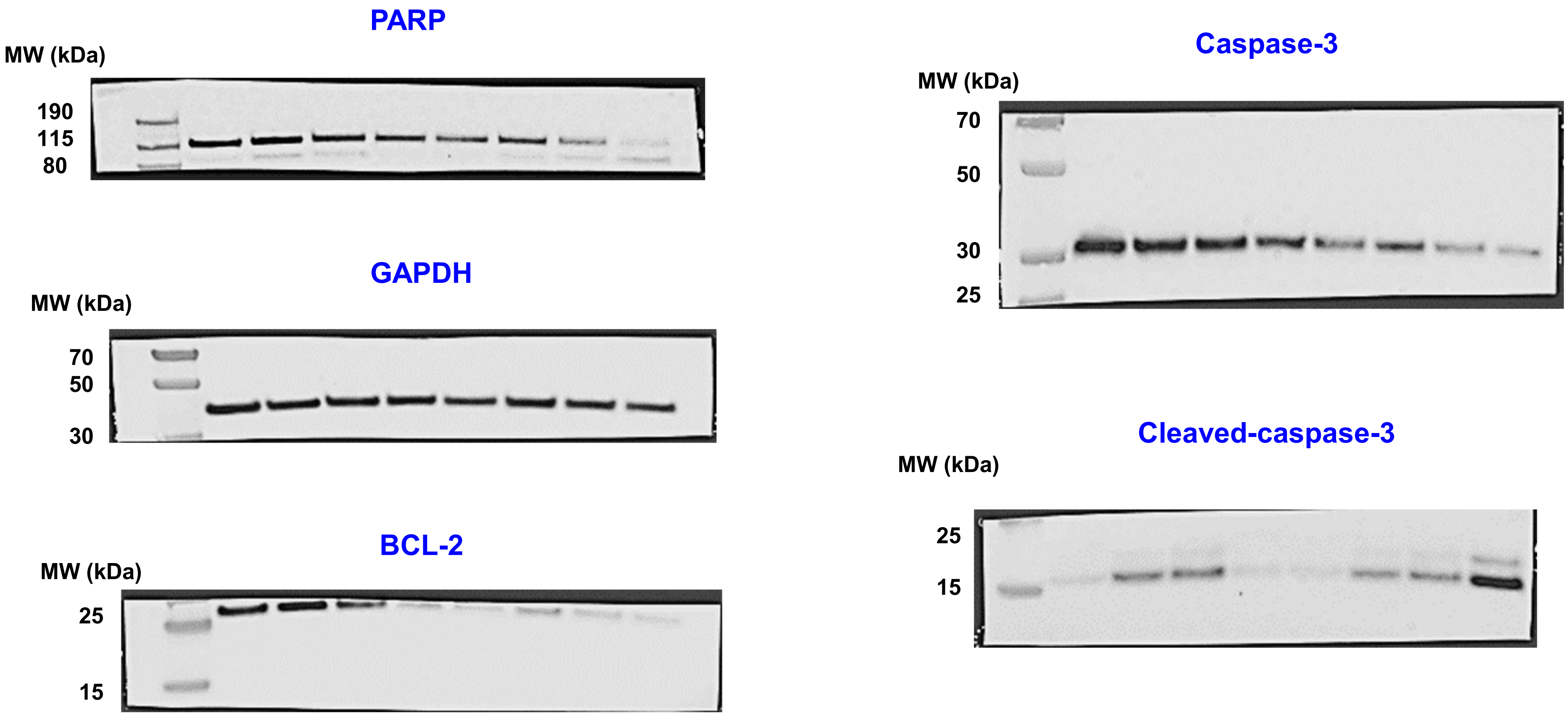

Figure 9E KG-1 cells

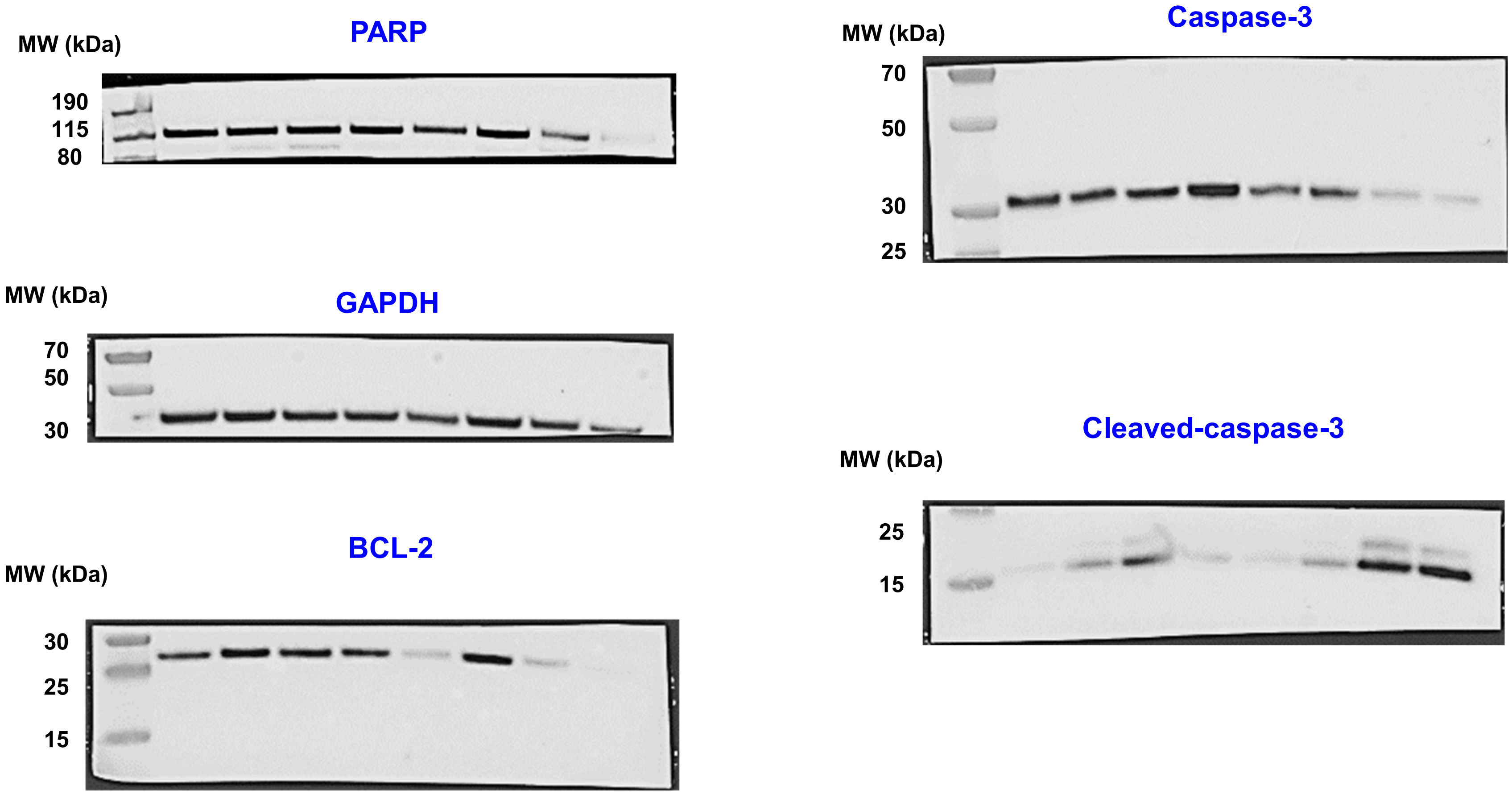

Figure S8 KG-1a cells

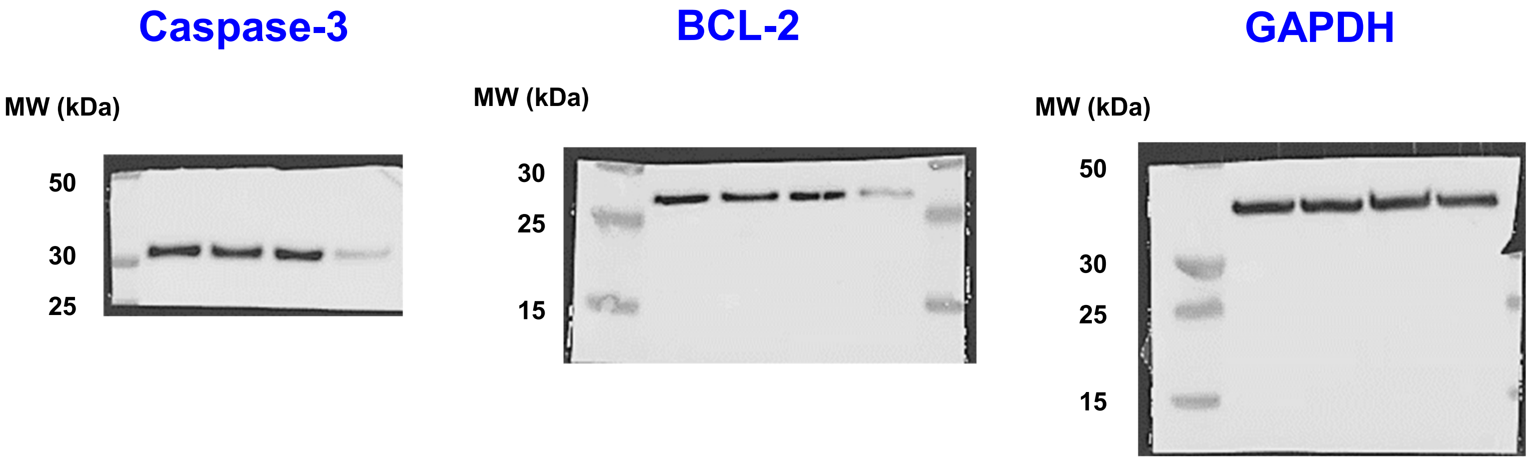

Supplement: Supplementary file 1 — Original WB [file 41420_2024_2148_MOESM1_ESM.pdf]
