# Supplementary material for: Bithionol eliminates acute myeloid leukaemia stem-like cells by suppressing NF-κB signalling and inducing oxidative stress, leading to apoptosis and ferroptosis
Source: Cell Death Discov. 2024 Aug 29;10:390. doi: 10.1038/s41420-024-02148-3 (PMC11362533; doi:10.1038/s41420-024-02148-3)
Supplement: Supplementary file 2 — SUPPLEMENTAL MATERIAL [file 41420_2024_2148_MOESM2_ESM.pdf]

Supplementary Material

Bithionol eliminates acute myeloid leukaemia stem-like cells by suppressing NF-κB signalling and inducing oxidative stress, leading to apoptosis and ferroptosis

Ingrid R. S. B. Dias[1], Rafaela G. A. Costa[1], Ana Carolina B. da C. Rodrigues[1], Suellen L. R. Silva[1], Maiara de S. Oliveira[1], Milena B. P. Soares[1,2], Rosane B. Dias[1,3,4], Ludmila F. Valverde[1,5], Clarissa A. Gurgel Rocha[1,3,6], Lauren V. Cairns[7], Ken I. Mills[7], Daniel P. Bezerra[1,*]

[1]Gonçalo Moniz Institute, Oswaldo Cruz Foundation (IGM-FIOCRUZ/BA), Salvador, Bahia, 40296-710, Brazil.

[2]SENAI Institute for Innovation in Advanced Health Systems, SENAI CIMATEC, Salvador, BA, 41650-010, Brazil.

[3]Department of Propaedeutics, Faculty of Dentistry of the Federal University of Bahia (UFBA), Salvador, Bahia, 40301-155, Brazil.

[4]Department of Biological Sciences, State University of Feira de Santana, Feira de Santana, Bahia, 44036-900, Brazil.

[5]Department of Dentistry, Federal University of Sergipe, Lagarto, Sergipe, 49400-000, Brazil.

[6]Center for Biotechnology and Cell Therapy, D'Or Institute for Research and Education (IDOR), Salvador, Bahia, 41253-190, Brazil.

[7]Patrick G Johnston Centre for Cancer Research, Queen's University Belfast, Belfast BT9 7AE, Northern Ireland, UK.

*Corresponding authors:

D. P. Bezerra, E-mail: daniel.bezerra@fiocruz.br

Phone number: + 55 71 3176 2272.

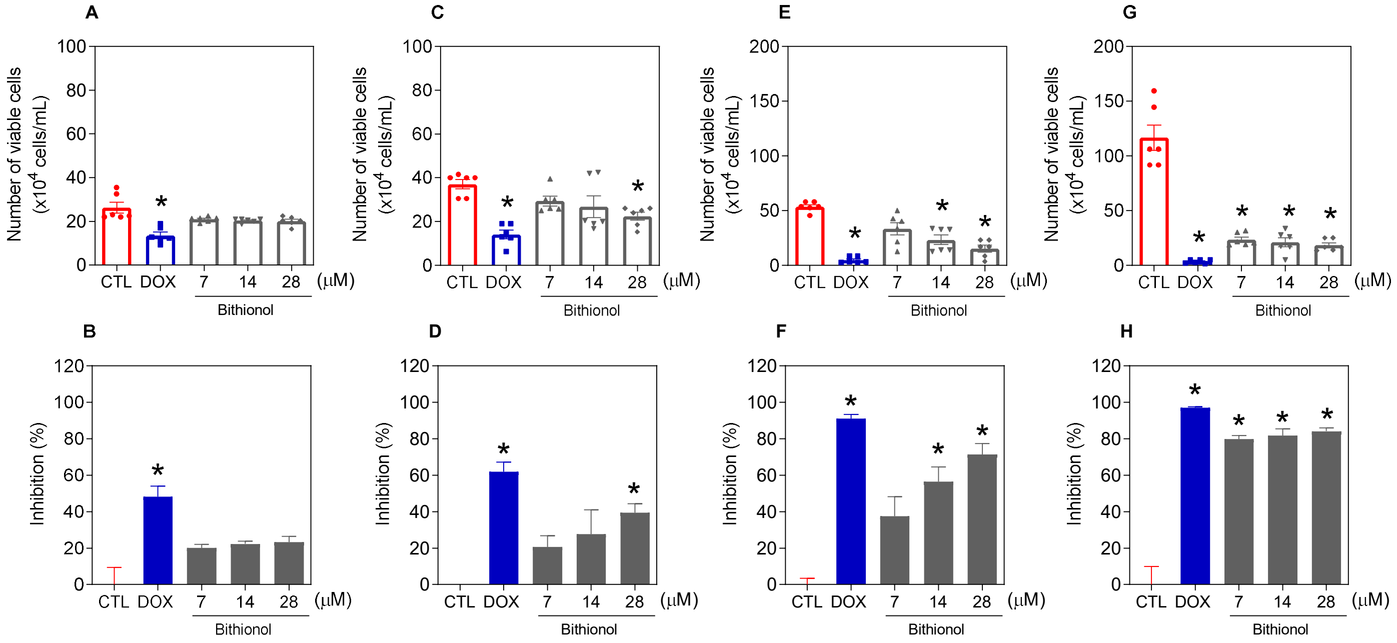

Figure S1. Trypan blue exclusion assay after 12 (**A** and **B**), 24 (**C** and **D**), 48 (**E** and **F**) and 72 (**G** and **H**) h of treatment with bithionol in KG-1a cells. Vehicle (0.2% DMSO) was used as a negative control (CTL), and doxorubicin (DOX, 1 μM) was used as a positive control. The data are shown as the mean ± S.E.M. of three biological replicates carried out in duplicate. * $p < 0.05$ compared with CTL by one-way ANOVA followed by Dunnett's multiple comparisons test.

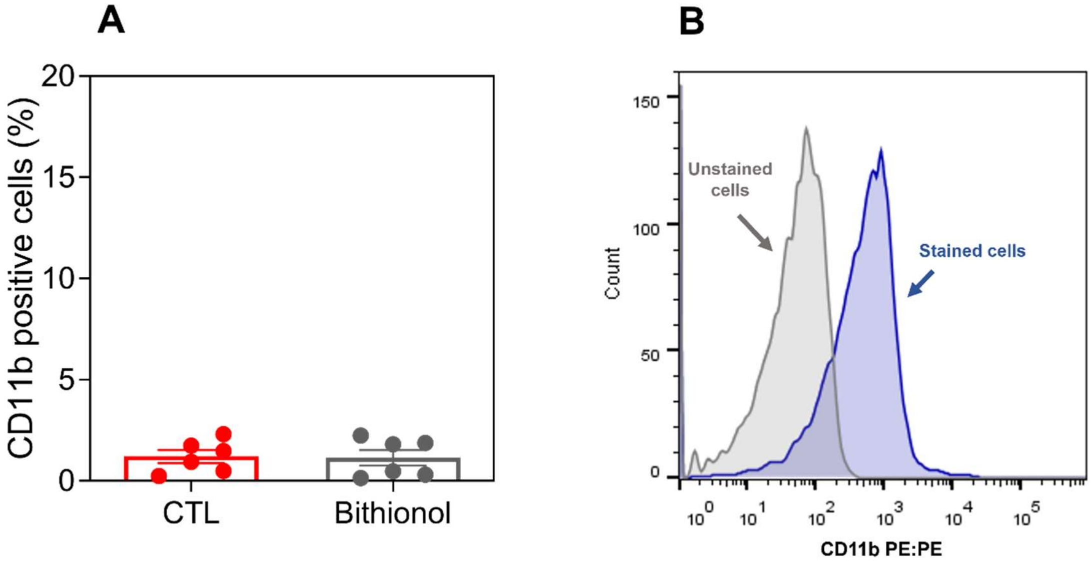

Figure S2. (**A**) Immunophenotypic analysis of the myeloid differentiation marker CD11b in KG-1a cells treated with 28 μM bithionol for 24 h. Vehicle (0.2% DMSO) was used as a negative control (CTL). The data are shown as the mean ± S.E.M. of three biological replicates carried out in duplicate. (**B**) Representative flow cytometry histogram of PBMCs stained with the anti-CD11b antibody, which was used as a positive control.

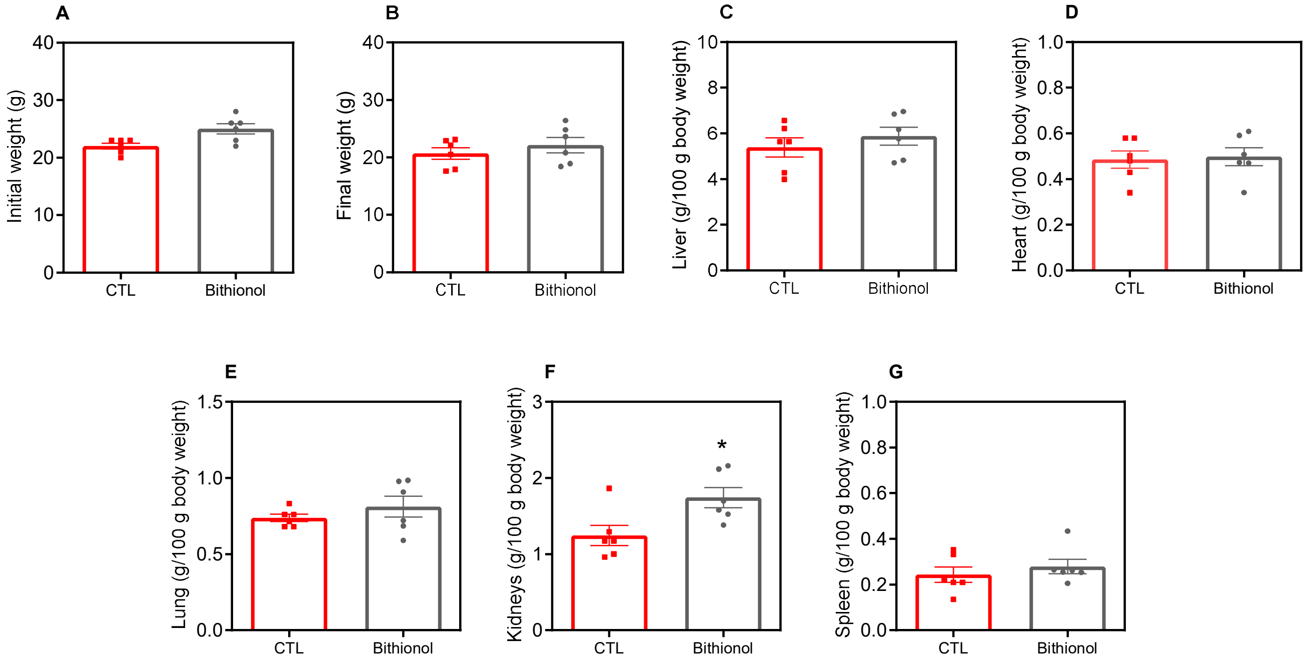

Figure S3. Effects of bithionol treatment on the body weight (**A** and **B**) and relative organ weight (**C, D, E, F,** and **G**) of NOD. Cg-*Prkdc*^{scid} *Il2rg*^{tm1Wjl}/SzJ (NSG) mice with AML KG-1a cell xenografts. The treatments (50 mg/kg bithionol) were injected into the mice intraperitoneally every day for two weeks. The negative control (CTL) was treated with the vehicle (5% DMSO) used for diluting bithionol. The data are shown as the mean ± S.E.M. from 6 animals. * $P < 0.05$ compared with CTL by Student's *t* test.

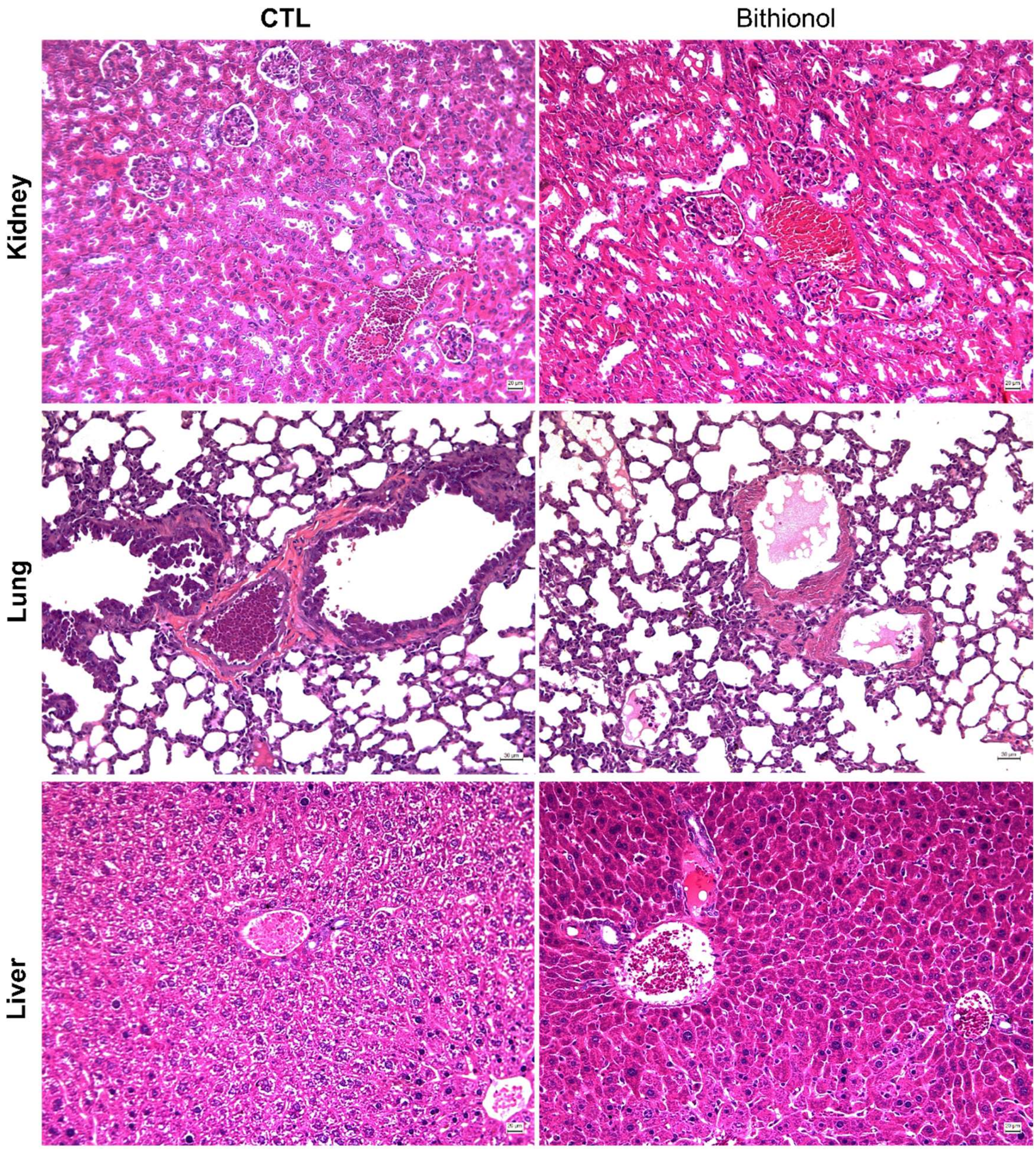

Figure S4. Representative photomicrographs of the kidneys, lungs, and livers of NOD. Cg-*Prkdc*^{scid} *Il2rg*^{tm1Wjl}/SzJ (NSG) mice with AML KG-1a cell xenografts that were treated with 50 mg/kg bithionol. The negative control (CTL) was treated with the vehicle (5% DMSO) used for diluting bithionol. The treatments were injected into the mice intraperitoneally every day for two weeks. Scale bar = 20 µm. Histological analysis of the organs was performed via optical microscopy, and histopathological changes were categorized as mild, moderate, or severe.

Renal architecture was preserved in animals from the control and bithionol groups. Among the histological changes observed in the group treated with bithionol, vascular hyperaemia, which ranged from moderate to severe, and specific areas of polymorphonuclear infiltration, coagulation necrosis of the renal cortex tubules and fibrosis were observed. Furthermore, a decrease in Bowman's space, caused by slight glomerular hyalinization, was observed in the kidneys of the animals. The lungs of the animals treated with bithionol had partially preserved architecture, mainly due to thickening of the alveolar septa and atelectasis. Vascular hyperaemia ranged from mild to severe, and focal areas of haemorrhage and polymorphonuclear cell infiltration were observed in the lungs of all groups. The architecture of the parenchyma and hepatic portal system was partially preserved in the control and bithionol-treated animals. Among the histopathological changes observed in animals treated with bithionol, hydropic degeneration, which varied from mild to moderate, moderate vascular hyperaemia and specific areas with hepatocytes in coagulation necrosis and infiltration of mononuclear and polymorphonuclear cells, was highlighted. The hearts of the animals did not significantly change (data not shown).

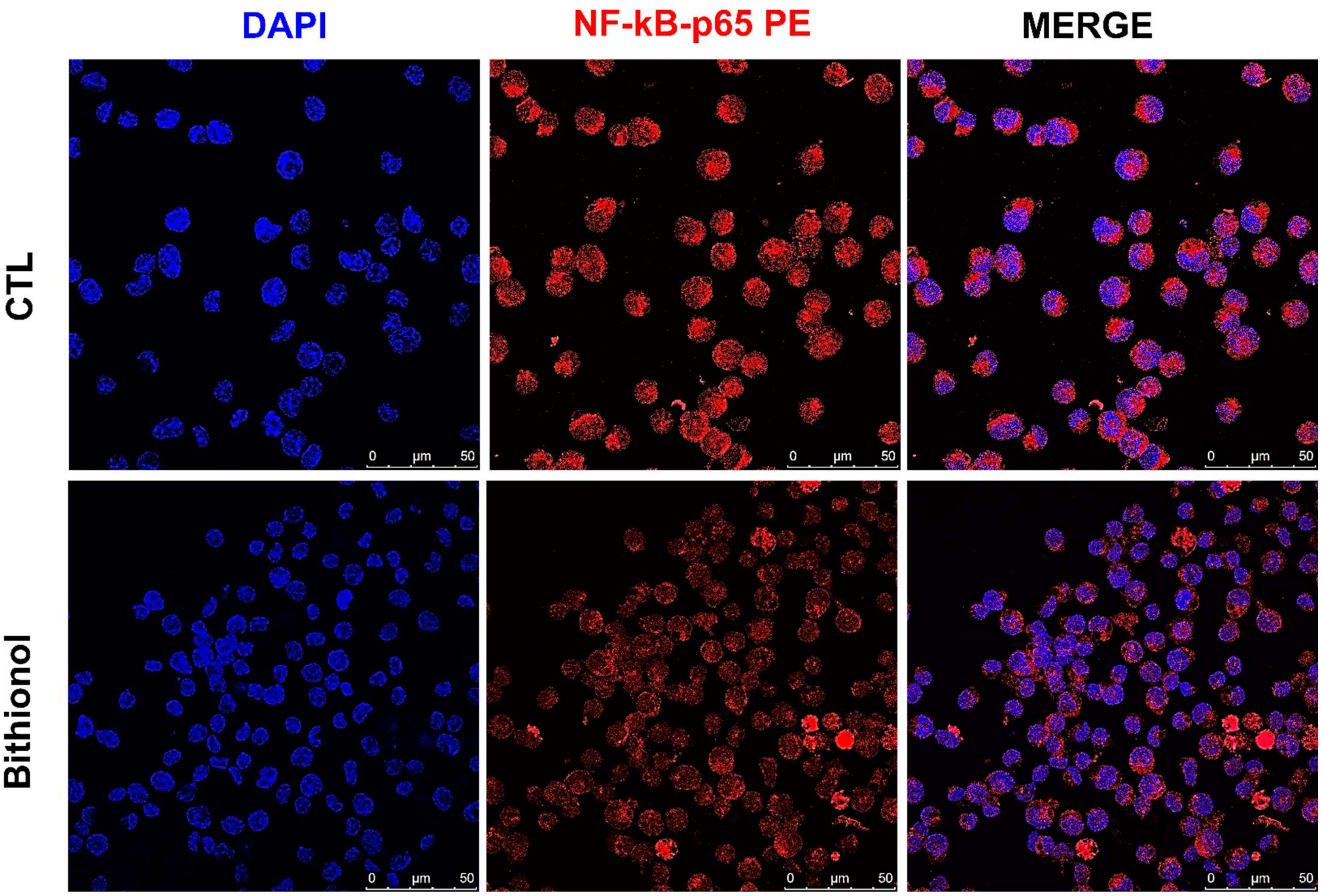

Figure S5. Uncropped immunofluorescence images of NF-κB p65 in KG-1a cells after 24 h of treatment with 28 μM bithionol (as shown in **Figure 4F**). Scale bar = 50 μm.

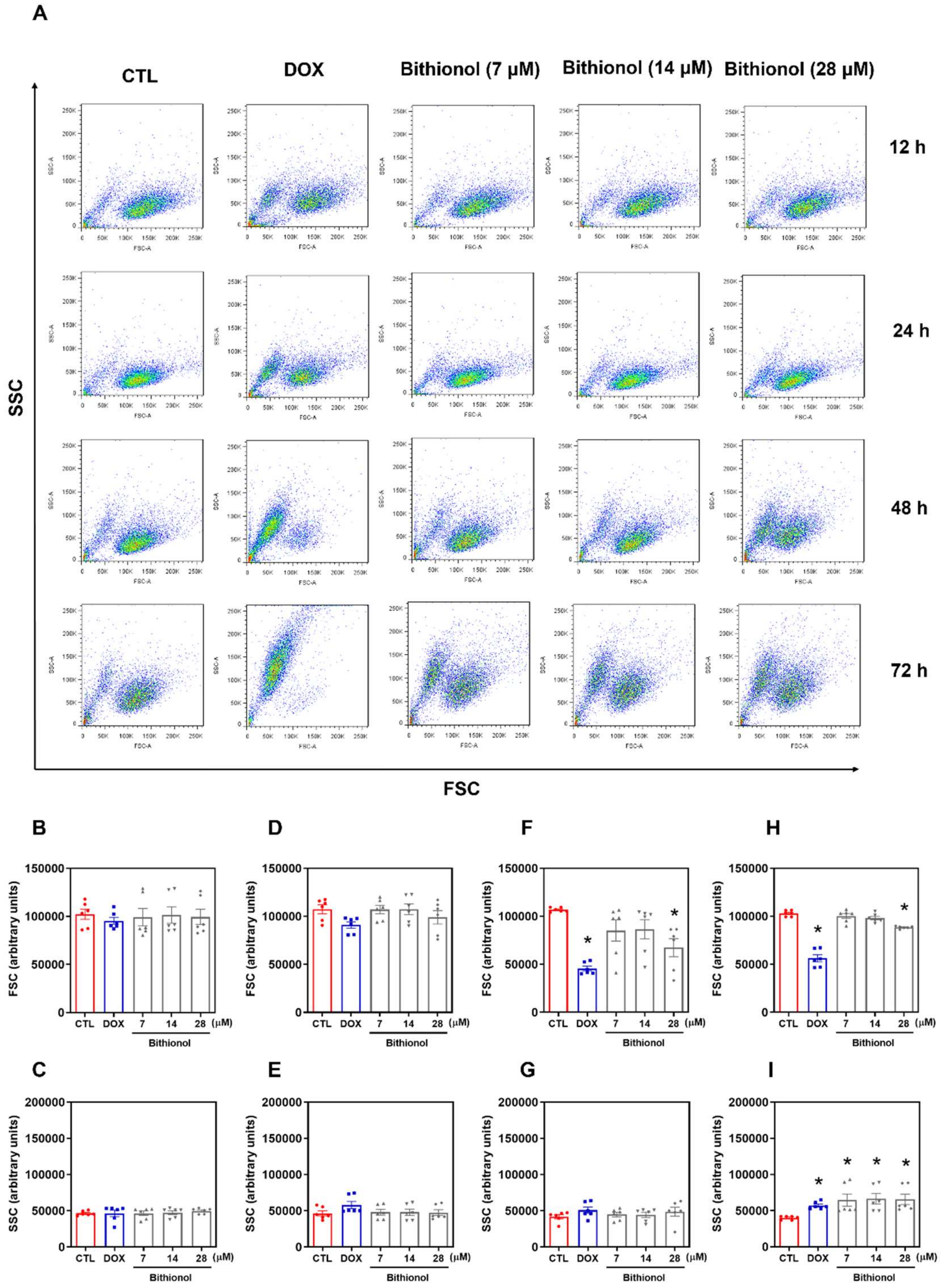

Figure S6. Effect of bithionol on the morphology of KG-1a cells, as evaluated by light-scattering features (FSC: forward scatter; SSC: side scatter) detected by flow cytometry after 12 (**A, B,** and **C**), 24 (**A, D,** and **E**), 48 (**A, F,** and **G**) and 72 (**A, H,** and **I**) h of treatment. The vehicle (0.2% DMSO) was used as a control (CTL), and doxorubicin (DOX, 1 μM) was used as a positive control. The data are shown as the mean ± S.E.M. of three biological replicates carried out in duplicate. * $P < 0.05$ compared with CTL by one-way ANOVA followed by Dunnett's multiple comparisons test.

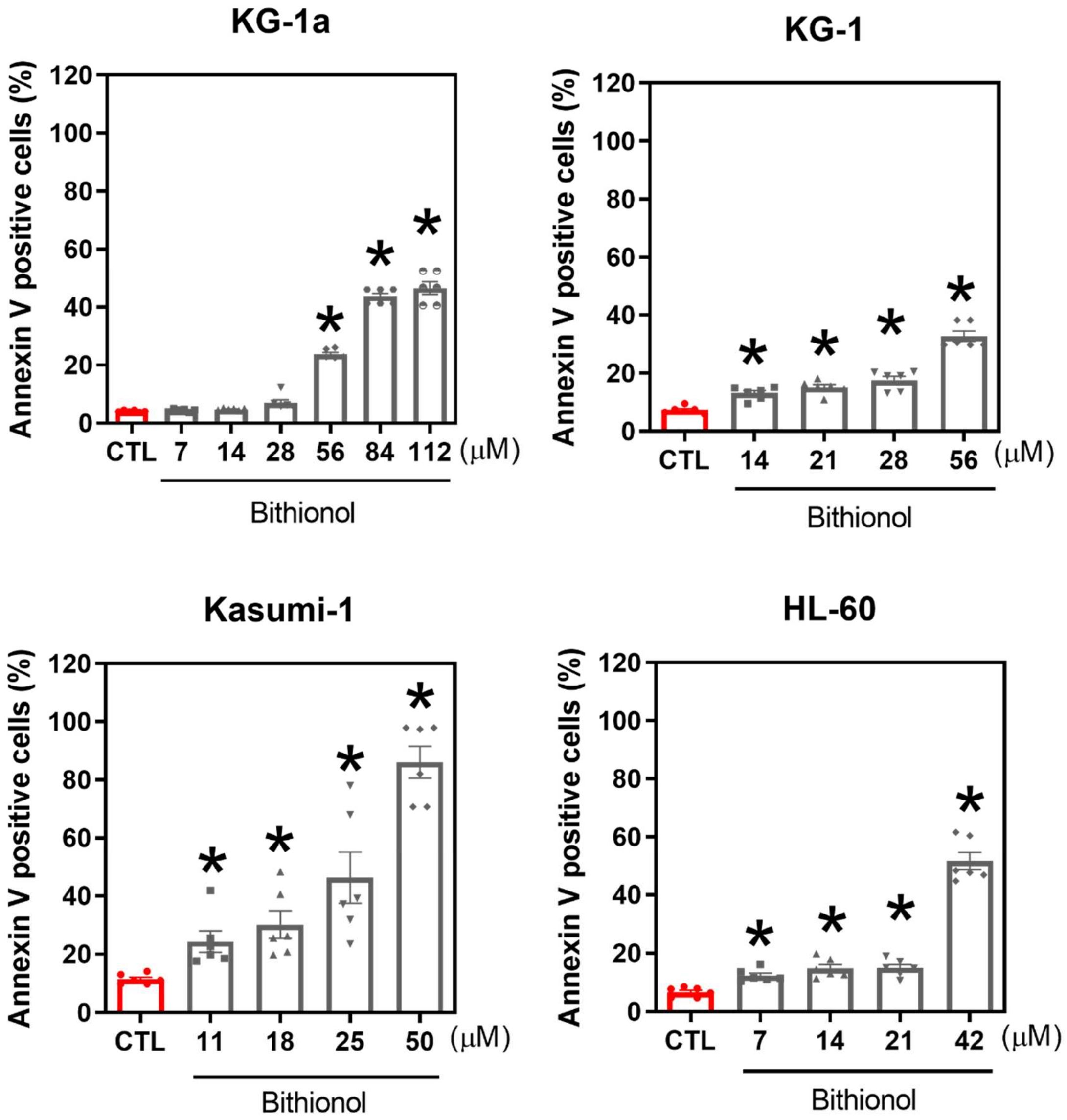

Figure S7. Phosphatidylserine externalization induced by bithionol in KG-1a, KG-1, Kasumi-1, and HL-60 cells after 72 h of treatment. The vehicle (0.2% DMSO) was used as a negative control (CTL). The data are shown as the mean ± S.E.M. of three biological replicates carried out in duplicate. * $p < 0.05$ compared with CTL by one-way ANOVA followed by Dunnett's multiple comparisons test.

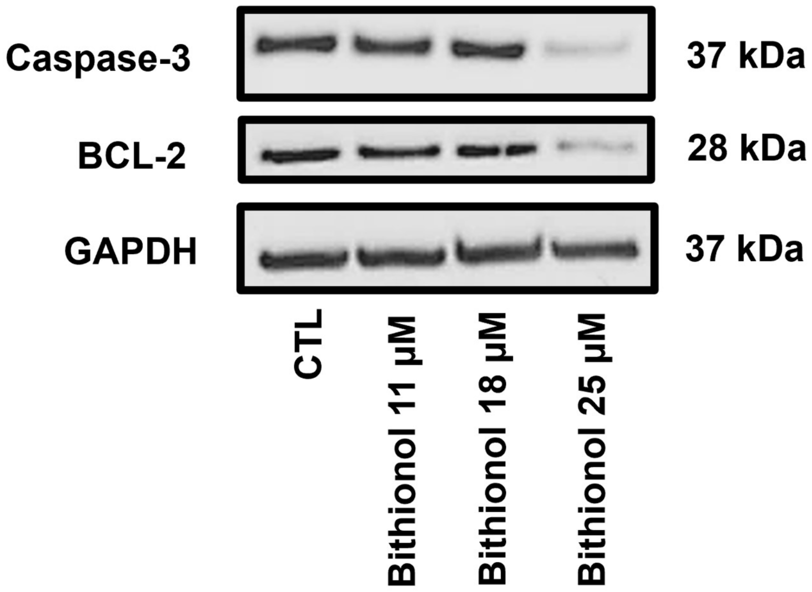

Figure S8. Western blot of caspase-3 and BCL-2 proteins from KG-1a cells treated with bithionol for 72 h. Vehicle (0.2% DMSO) was used as a negative control (CTL). GAPDH was used as an internal control.

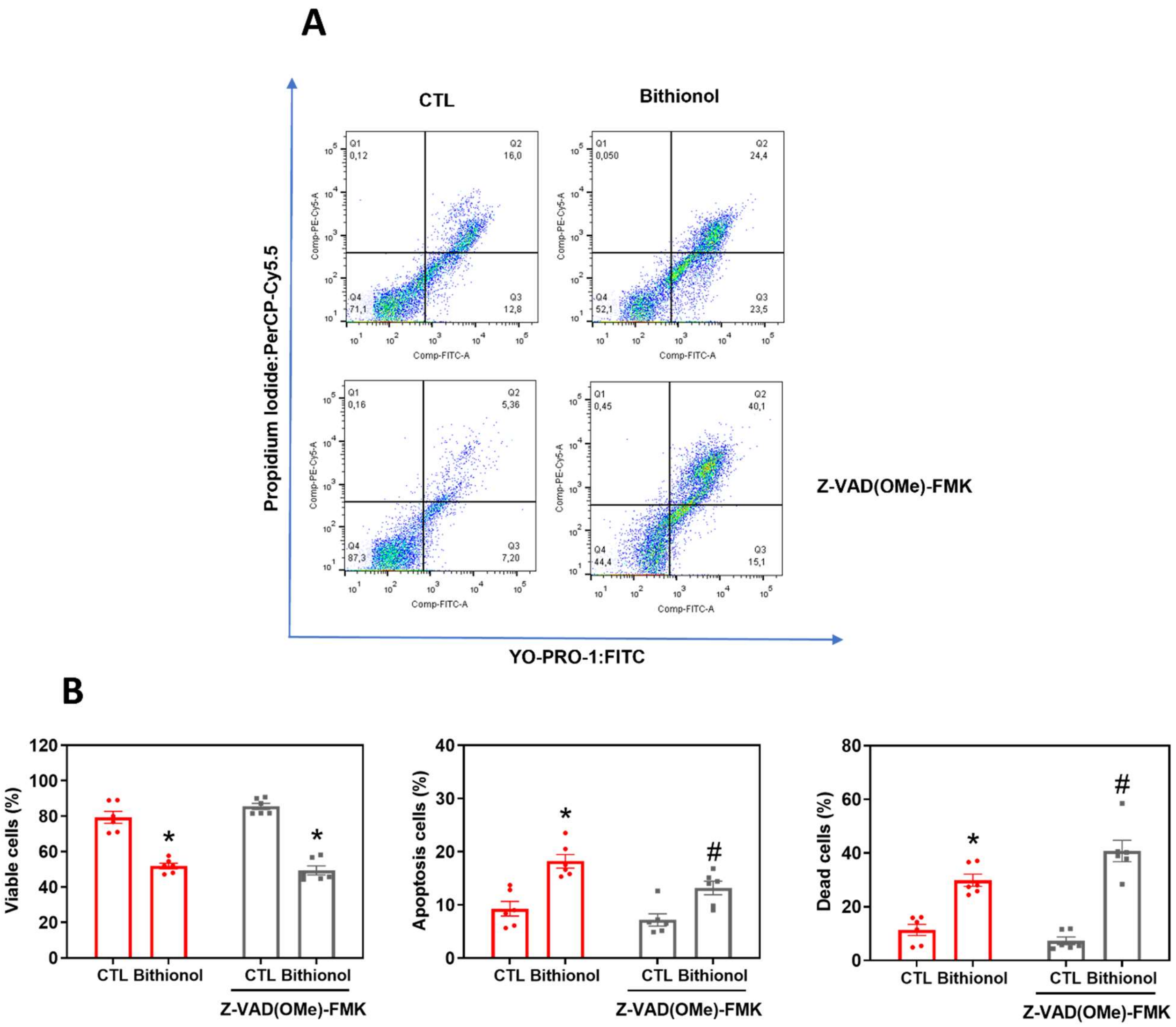

Figure S9. Effect of the pancaspase inhibitor Z-VAD(OMe)-FMK on bithionol-induced apoptosis in KG-1a cells. (**A**) Representative flow cytometry dot plot graphs. (**B**) Quantification of viable, apoptotic and dead cells. The cells were pretreated for 2 h with 50 μM Z-VAD(OMe)-FMK and then incubated with 28 μM bithionol for 72 h. Vehicle (0.2% DMSO) was used as a negative control (CTL). The data are shown as the mean ± S.E.M. of three biological replicates carried out in duplicate. * p < 0.05 compared with CTL by Student's t test. # p < 0.05 compared with the respective treatment without inhibitor by Student's t test.

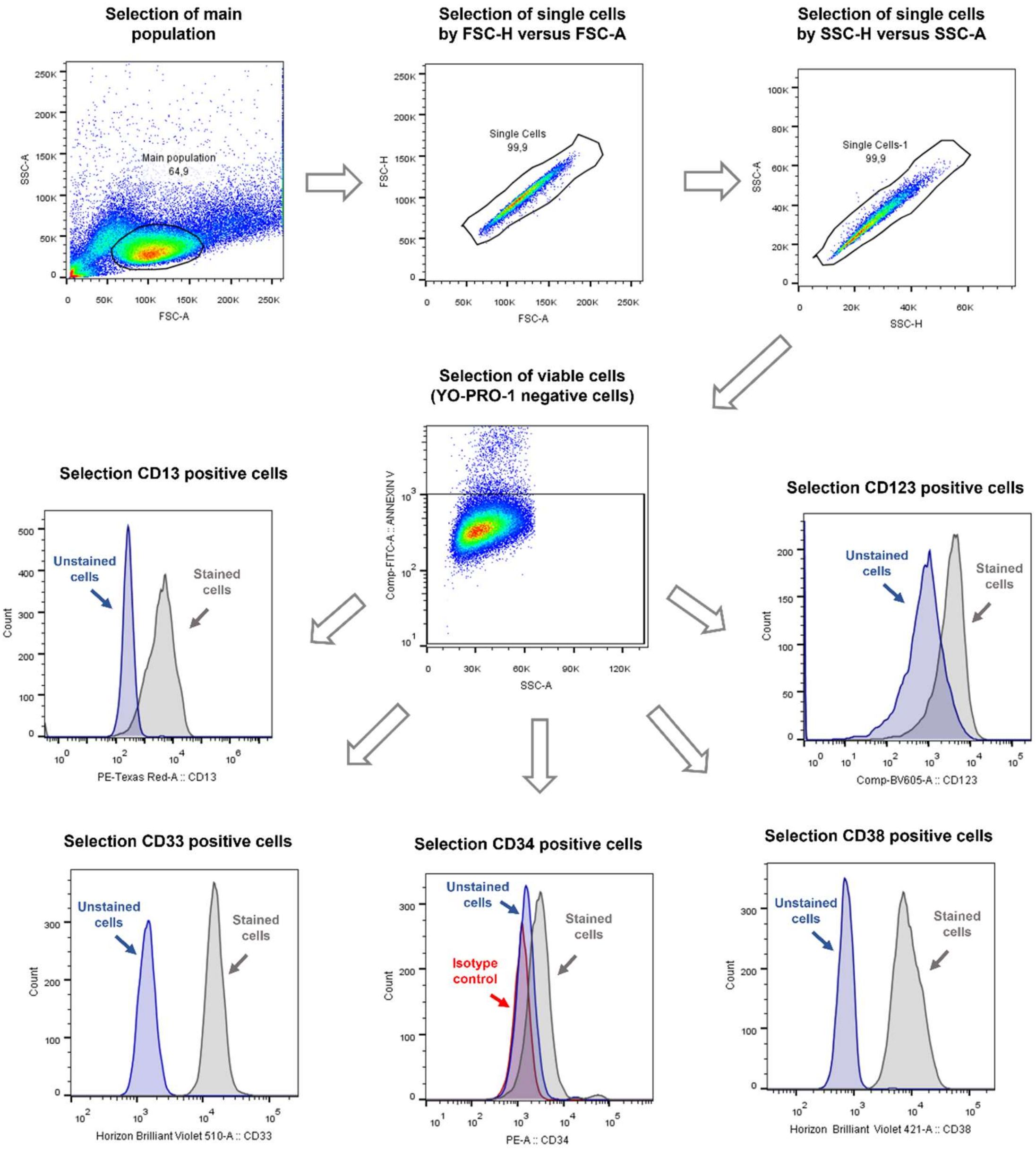

Figure S10. Representative gating strategy for the immunophenotyping of KG-1a cells by flow cytometry. PE mouse anti-human CD34, BV421 mouse anti-human CD38, BV605 mouse anti-human CD123, PE-CF594 mouse anti-human CD13 and BV510 mouse anti-human CD33 antibodies were used. A PE mouse IgG1κ isotype control was used as an isotype control, and YO-PRO-1 was used to select viable cells.

Table S1. Cytotoxicity of bithionol

Cells	IC_{50} and 95% CI (μM)	
	**Bithionol**	**DOX**
Haematological cancer cells		
Jurkat	19.01	0.04
	14.49 – 24.95	0.01 – 0.10
MOLM-13	26.99	0.01
	17.39 – 41.88	0.01 – 0.02
KG-1	20.50	0.30
	22.72 – 29.35	0.20 – 0.42
KG-1a	14.43	0.54
	4.08 – 21.05	0.23 – 1.25
Kasumi-1	18.82	3.53
	13.45 – 26.29	2.28 – 5.48
NB4	19.52	0.12
	13.44 – 28.36	0.08 – 0.19
HL-60	19.46	0.27
	15.18 – 24.95	0.19 – 0.40
K-562	32.40	1.24
	24.99 – 41.99	0.69 – 2.24
THP-1	33.58	0.21
	27.83 – 40.53	0.13 – 0.35
PL-21	17.61	0.04
	12.44 – 24.91	0.03 – 0.05

MV4-11	16.74	0.01
	8.00 – 34.97	0.01 – 0.02
Solid cancer cells		
MCF-7	38.81	1.23
	27.76 – 54.26	0.78 - 1,91
MDA-MB-231	32.51	0.55
	27.14 – 38.94	0.24 – 1.25
4T1	47.02	1.53
	33.01 – 66.98	1.08 – 2.17
HCT116	16.74	0.07
	12.56 – 22.33	0.04 – 0.13
B16-F10	27.31	0.09
	18.91 – 39.43	0.05 – 0.15
A375	27.50	0.24
	23.27 – 32.50	0.20 – 0.30
HepG2	26.59	0.26
	17.53 – 40.32	0.15 – 0.44
OVCAR-3	24.92	1.54
	18.21 – 34.11	0.94 – 2.53
U-87 MG	35.51	0.38
	29.24 – 43.14	0.25 – 0.57
A549	34.22	2.24
	23.72 – 49.37	1.83 – 2.75
PANC-1	16.94	1.40
	13.49 – 21.28	0.93 – 2.11

DU 145	49.26<br>38.40 – 63.20	0.34<br>0.24 – 0.48
HSC-3	35.44<br>27.29 – 46.02	0.43<br>0.31 – 0.58
CAL27	20.76<br>14.87 – 28.98	0.55<br>0.39 – 0.78
SCC-4	22.17<br>18.08 – 27.18	0.46<br>0.18 – 1.15
SCC-9	>70	1.13<br>0.64 – 2.00
SCC-25	>70	1.30<br>0.71 – 2.37
Noncancerous cells		
PBMC	59.63<br>54.07 – 65.75	0.97<br>0.59 – 1.59
BJ	53.36<br>40.81 – 69.76	1.63<br>0.17 – 3.83
MRC-5	48.36<br>37.10 – 63.04	1.32<br>0.83 – 2.11

These data were obtained via nonlinear regression from three biological replicates carried out in duplicate after 72 h of incubation. Doxorubicin (DOX) was used as a positive control.

Table S2. Selectivity indices obtained

Cancer cells	Noncancerous cells					
	PBMC		BJ		MRC-5	
	Bithionol	DOX	Bithionol	DOX	Bithionol	DOX
Haematological cancer cells						
Jurkat	3.1	24.3	2.8	40.8	2.5	33.0
MOLM-13	2.2	97.0	2.0	163.0	1.8	132.0
KG-1	2.9	3.2	2.6	5.4	2.4	4.4
KG-1a	4.1	1.8	3.7	3.0	3.4	2.4
Kasumi-1	3.2	0.3	2.8	0.5	2.6	0.4
NB4	3.1	8.1	2.7	13.6	2.5	11.0
HL-60	3.1	3.6	2.7	6.0	2.5	4.9
K-562	1.8	0.8	1.6	1.3	1.5	1.1
THP-1	1.8	4.6	1.6	7.8	1.4	6.3
PL-21	3.4	24.3	3.0	40.8	2.7	33.0
MV4-11	3.6	97.0	3.2	163.0	2.9	132.0
Solid cancer cells						
MCF-7	1.5	0.8	1.4	1.3	1.2	1.1
MDA-MB-231	1.8	1.8	1.6	3.0	1.5	2.4
4T1	1.3	0.6	1.1	1.1	1.0	0.9
HCT116	3.6	13.9	3.2	23.3	2.9	18.9
B16-F10	2.2	10.8	2.0	18.1	1.8	14.7
A375	2.2	4.0	1.9	6.8	1.8	5.5

HepG2	2.2	3.7	2.0	6.3	1.8	5.1
OVCAR-3	2.4	0.6	2.1	1.1	1.9	0.9
U-87 MG	1.7	2.6	1.5	4.3	1.4	3.5
A549	1.7	0.4	1.6	0.7	1.4	0.6
PANC-1	3.5	0.7	3.1	1.2	2.9	0.9
DU 145	1.2	2.9	1.1	4.8	1.0	3.9
HSC-3	1.7	2.3	1.5	3.8	1.4	3.1
CAL27	2.9	1.8	2.6	3.0	2.3	2.4
SCC-4	2.7	2.1	2.4	3.5	2.2	2.9
SCC-9	N.d.	0.9	N.d.	1.4	N.d.	1.2
SCC-25	N.d.	0.7	N.d.	1.3	N.d.	1.0

The data were calculated via the following formula: selectivity indices = IC_{50} [noncancerous cells]/IC_{50} [cancer cells]. N.d. = not determined.

Table S3. The effect of bithionol on gene expression in KG-1a cells

Function/Assay ID	Gene Symbol	Gene Name	RQ	
			CTL	Bithionol
**NFkB pathway**				
Hs00765730_m1	*NFKB1*	nuclear factor kappa B subunit 1	1.00	1.564
Hs00174517_m1	*NFKB2*	nuclear factor kappa B subunit 2	1.00	1.658
Hs00153283_m1	*NFKBIA*	NFKB inhibitor alpha	1.00	1.818
Hs00182115_m1	*NFKBIB*	NFKB inhibitor beta	1.00	0.959
Hs00153294_m1	*RELA*	RELA proto-oncogene, NF-kB subunit	1.00	1.313
Hs00232399_m1	*RELB*	RELB proto-oncogene, NF-kB subunit	1.00	4.045
**WNT/β-catenin pathway**				
Hs00181051_m1	*APC*	APC, WNT signalling pathway regulator	1.00	1.554
Hs00793391_m1	*CSNK1A1*	casein kinase 1 alpha 1	1.00	1.482
Hs00170025_m1	*CTNNB1*	catenin beta 1	1.00	0.894
Hs00275656_m1	*GSK3B*	glycogen synthase kinase 3 beta	1.00	1.714
Hs00228741_m1	*WNT10A*	Wnt family member 10A	n.d.	n.d.
Hs00559664_m1	*WNT10B*	Wnt family member 10B	1.00	0.376
Hs00257131_m1	*WNT2B*	Wnt family member 2B	n.d.	n.d.
Hs00362452_m1	*WNT6*	Wnt family member 6	n.d.	n.d.
**Hedgehog pathway**				

Hs00368306_m1	*DHH*	desert hedgehog	n.d.	n.d.
Hs00171790_m1	*GLI1*	GLI family zinc finger 1	1.00	0.696
Hs00257977_m1	*GLI2*	GLI family zinc finger 2	n.d.	n.d.
Hs00181117_m1	*PTCH1*	patched 1	1.00	1.025
Hs00179843_m1	*SHH*	sonic hedgehog	n.d.	n.d.
Hs00170665_m1	*SMO*	smoothened, frizzled class receptor	n.d.	n.d.
Hs00171981_m1	*SUFU*	SUFU negative regulator of hedgehog signalling	1.00	1.512
**NOTCH pathway**				
Hs00194509_m1	*DLL1*	delta like canonical Notch ligand 1	1.00	n.d.
Hs01085096_m1	*DLL3*	delta like canonical Notch ligand 3	1.00	n.d.
Hs00164982_m1	*JAG1*	jagged 1	1.00	1.560
Hs00171432_m1	*JAG2*	jagged 2	1.00	n.d.
Hs01062014_m1	*NOTCH1*	notch 1	1.00	1.457
Hs01050702_m1	*NOTCH2*	notch 2	1.00	1.189
**EGFR pathway**				
Hs01099999_m1	*EGF*	epidermal growth factor	1.00	n.d.
Hs01076078_m1	*EGFR*	epidermal growth factor receptor	n.d.	n.d.
Hs00364282_m1	*KRAS*	KRAS proto-oncogene, GTPase	1.00	1.322
Hs01046830_m1	*MAPK1*	mitogen-activated protein kinase 1	1.00	1.448

Hs00234119_m1	*RAF1*	Raf-1 proto-oncogene, serine/threonine kinase	1.00	0.982
Hs00269660_s1	*RHOB*	ras homologue family member B	n.d.	n.d.
**JAK/STAT pathway**				
Hs01026983_m1	*JAK1*	Janus kinase 1	1.00	1.370
Hs01078136_m1	*JAK2*	Janus kinase 2	1.00	1.202
Hs00169663_m1	*JAK3*	Janus kinase 3	n.d.	n.d.
Hs01013989_m1	*STAT1*	signal transducer and activator of transcription 1	1.00	1.831
Hs00374280_m1	*STAT3*	signal transducer and activator of transcription 3	1.00	2.040
Hs00273500_m1	*STAT5B*	signal transducer and activator of transcription 5B	1.00	1.337
Hs00598625_m1	*STAT6*	signal transducer and activator of transcription 6	1.00	1.175
**PI3K/AKT/MTOR pathway**				
Hs00178289_m1	*AKT1*	AKT serine/threonine kinase 1	1.00	0.881
Hs01086102_m1	*AKT2*	AKT serine/threonine kinase 2	1.00	1.519
Hs00234508_m1	*MTOR*	mechanistic target of rapamycin	1.00	1.523
Hs00904054_m1	*PIK3C2A*	phosphatidylinositol-4-phosphate 3-kinase catalytic subunit type 2 alpha	1.00	1.624

Hs00176908_m1	*PIK3C3*	phosphatidylinositol 3-kinase catalytic subunit type 3	1.00	1.726
Hs00907957_m1	*PIK3CA*	phosphatidylinositol-4,5-bisphosphate 3-kinase catalytic subunit alpha	1.00	2.400
Hs02621230_s1	*PTEN*	phosphatase and tensin homologue	1.00	1.279
**TGF-beta/SMAD pathway**				
Hs01054576_m1	*FOXO1*	forkhead box O1	1.00	2.193
Hs00183425_m1	*SMAD2*	SMAD family member 2	1.00	3.347
Hs00929647_m1	*SMAD4*	SMAD family member 4	1.00	1.230
Hs00178696_m1	*SMAD7*	SMAD family member 7	1.00	1.521
Hs00998133_m1	*TGFB1*	transforming growth factor beta 1	1.00	1.152
Hs00234244_m1	*TGFB2*	transforming growth factor beta 2	1.00	2.435
Hs01086000_m1	*TGFB3*	transforming growth factor beta 3	1.00	1.504
**PPAR pathway**				
Hs00947536_m1	*PPARA*	peroxisome proliferator activated receptor alpha	1.00	1.172
Hs04187066_g1	*PPARD*	peroxisome proliferator activated receptor delta	1.00	2.224
Hs01115513_m1	*PPARG*	peroxisome proliferator activated receptor gamma	1.00	5.457
Hs01016719_m1	*PPARGC1A*	PPARG coactivator 1 alpha	n.d.	n.d.
Hs00991677_m1	*PPARGC1B*	PPARG coactivator 1 beta	1.00	1.552

**Oxidative stress**				
Hs00943350_g1	*GSTP1*	glutathione S-transferase pi 1	1.00	0.634
Hs00178247_m1	*OXSR1*	oxidative stress responsive 1	1.00	0.965
Hs00167309_m1	*SOD2*	superoxide dismutase 2, mitochondrial	1.00	0.783
Hs01555214_g1	*TXN*	thioredoxin	1.00	1.163
Hs00917067_m1	*TXNRD1*	thioredoxin reductase 1	1.00	1.707
**Apoptosis**				
Hs00559441_m1	*APAF1*	apoptotic peptidase activating factor 1	1.00	1.591
Hs00188930_m1	*BAD*	BCL2 associated agonist of cell death	1.00	0.612
Hs00180269_m1	*BAX*	BCL2 associated X, apoptosis regulator	1.00	1.299
Hs99999018_m1	*BCL2*	BCL2, apoptosis regulator	1.00	0.886
Hs00609632_m1	*BID*	BH3 interacting domain death agonist	1.00	0.961
Hs00234387_m1	*CASP3*	caspase 3	1.00	1.418
Hs00169152_m1	*CASP7*	caspase 7	1.00	1.344
Hs00219876_m1	*DIABLO*	diablo IAP-binding mitochondrial protein	1.00	0.801
Hs00538709_m1	*FADD*	Fas associated via death domain	1.00	1.424
Hs00531110_m1	*FAS*	Fas cell surface death receptor	1.00	1.276

Hs00242302_m1	*PARP1*	poly(ADP-ribose) polymerase 1	1.00	0.662
**Autophagy**				
Hs01047860_g1	*ATG12*	autophagy related 12	1.00	1.118
Hs00223937_m1	*ATG3*	autophagy related 3	1.00	0.889
Hs00169468_m1	*ATG5*	autophagy related 5	1.00	0.847
Hs00186838_m1	*BECN1*	beclin 1	1.00	0.999
Hs01076567_g1	*MAP1LC3A*	microtubule associated protein 1 light chain 3 alpha	1.00	n.d.
Hs00797944_s1	*MAP1LC3B*	microtubule associated protein 1 light chain 3 beta	1.00	5.967
**Necroptosis**				
Hs00169407_m1	*RIPK1*	receptor interacting serine/threonine kinase 1	1.00	1.330
Hs01572686_m1	*RIPK2*	receptor interacting serine/threonine kinase 2	1.00	1.653
Hs01011177_g1	*RIPK3*	receptor interacting serine/threonine kinase 3	1.00	1.003
**Epithelial–mesenchymal transition**				
Hs00170423_m1	*CDH1*	cadherin 1	n.d.	n.d.
Hs00195591_m1	*SNAI1*	snail family transcriptional repressor 1	1.00	0.929
Hs00950344_m1	*SNAI2*	snail family transcriptional repressor 2	n.d.	n.d.

Hs01018996_m1	*SNAI3*	snail family transcriptional repressor 3	1.00	0.813
Hs00361186_m1	*TWIST1*	twist family bHLH transcription factor 1	1.00	0.289
Hs00185584_m1	*VIM*	vimentin	1.00	2.183
Hs00232783_m1	*ZEB1*	zinc finger E-box binding homeobox 1	1.00	1.654
Hs00207691_m1	*ZEB2*	zinc finger E-box binding homeobox 2	1.00	2.064

KG-1a cells were treated with 28 μM bithionol for 12 h. The negative control (CTL) was treated with the vehicle (0.2% DMSO) used for diluting bithionol. After treatment, total RNA was isolated and reverse transcribed. Gene expression was detected via a TaqMan® array plate 96 plus fast (#4413256). The *GUSB*, *HPRT1* and *GAPDH* genes were used as endogenous genes for normalization. The values represent the relative quantitation (RQ) compared with the calibrator (cells treated with the negative control). The genes were upregulated if RQ ≥ 2 and downregulated if RQ ≤ 0.5. N.d. Not determined.

Table S4. Cell viability after 72 h of treatment with 48 drugs combined with bithionol

Drugs	Cell viability (%)							
	KG1-a		KG-1		Kasumi-1		HL-60	
	Single agents (2 nM)	Plus bithionol (28 μM)	Single agents (2 nM)	Plus bithionol (28 μM)	Single agents (2 nM)	Plus bithionol (28 μM)	Single agents (2 nM)	Plus bithionol (28 μM)
Anagrelide	82	77	88	60	76	60	85	64
Arsenic Trioxide	84	77	88	62	77	59	87	75
ATRA	82	70	81	55	76	57	85	64
Azacitidine	83	77	86	61	71	59	87	79
Cladribine	86	82	75	61	58	65	82	70
Crenolanib	85	81	88	61	77	61	87	75
Cyclophosphamide	85	86	88	69	78	64	86	57
Cytarabine	81	77	79	49	57	61	81	76
Daunorubicin	83	82	87	65	77	63	84	54
Decitabine	79	72	78	57	65	63	86	80
Doxorubicin	84	83	90	73	75	69	87	66
Enasidenib	83	80	88	69	75	58	85	72
Fludarabine	85	79	88	66	76	64	85	53
Gilteritinib	84	78	88	74	74	61	86	65
Hydroxyurea	84	83	88	74	78	64	86	44
Idarubicin	75	66	62	52	36	58	64	56

Imatinib	84	84	88	88	76	76	85	85
Ivosidenib	84	76	89	65	75	60	85	71
Lenolinomide	83	79	89	62	76	62	85	67
Midostaurin	84	78	88	51	73	61	85	68
Nivolumab	84	81	88	60	77	63	85	81
Pevonedistat	81	79	77	43	42	60	67	65
Pracinostat	72	60	77	60	28	59	86	68
Quizartinib	85	70	87	56	77	60	85	45
Rituximab	86	74	88	69	74	67	87	76
Ruxolitinib	85	76	87	57	77	61	87	73
Sorafenib	84	76	89	57	77	64	87	76
Thioguanine	73	78	80	68	73	59	87	75
Tipifarnib	73	74	72	70	73	66	87	76
Ubenimex	83	78	87	57	76	63	86	76
Venetoclax	80	68	86	48	76	70	86	76
Nilotinib	84	77	88	58	77	61	87	80
Dasatinib	84	78	88	61	77	65	86	81
Aspirin	84	75	88	64	77	59	84	78
Busulfan	84	77	88	69	76	66	85	76
4-Aminosalicylic Acid	84	77	88	66	76	59	85	80
Clofarabine	60	78	75	56	49	60	68	78
Dactinomycin	84	72	76	48	48	57	82	75

Donepezil·HCl	84	84	87	88	76	75	84	83
Gemcitabine·HCl	36	57	67	55	47	58	70	68
Paclitaxel (Taxol)	78	79	61	54	50	57	72	71
Vinblastine Sulfate	83	74	54	45	60	57	72	55
Irinotecan·HCl	84	82	88	45	75	62	85	66
Topotecan·HCl	39	62	69	48	36	59	71	75
Mebendazole	82	67	81	58	75	60	84	61
Olaparib	86	83	87	78	69	39	86	70
Vorinostat	75	61	67	50	38	56	87	64
Glasdegib	84	75	88	56	78	61	87	72
Control	85	84	88	49	77	64	85	76

Table S5. Cell viability after 72 h of treatment with 14 drugs combined with bithionol

Drugs	Cell viability (%)							
	KG1-a		KG-1		Kasumi-1		HL-60	
	Single agents (2 nM)	Plus bithionol (28 μM)	Single agents (2 nM)	Plus bithionol (28 μM)	Single agents (2 nM)	Plus bithionol (28 μM)	Single agents (2 nM)	Plus bithionol (28 μM)
Daunorubicin	82	81	86	84	80	81	89	87
Fludarabine	85	83	86	85	79	81	88	87
Hydroxyurea	82	80	87	84	79	81	88	88
Midostaurin	82	80	83	84	78	81	88	87
Pevonedistat	78	77	78	73	63	73	88	87
Quizartinib	82	79	86	84	82	79	87	88
Sorafenib	83	83	87	85	82	80	89	88
Ubenimex	80	79	85	85	78	81	89	88
Venetoclax	71	55	83	49	80	71	88	88
Irinotecan·HCl	82	81	84	84	78	82	88	88
Mebendazole	78	79	72	76	77	77	88	87
Olaparib	84	85	85	83	76	71	88	87
Vorinostat	37	43	63	77	44	57	87	87
Glasdegib	81	82	85	83	79	78	86	88
DMSO	82	83	86	82	80	78	88	88

Table S6. Combination index for bithionol plus venetoclax after 72 h of incubation

Cells	Venetoclax (nM)	Bithionol (μM)	Faction affected	Combination index
**KG-1a**	2	7	0.26303	0.79996
	2	14	0.52028	0.76288
	2	28	0.79485	0.6173
	10	7	0.3704	1.03945
	10	14	0.46294	1.22125
	10	28	0.71164	0.93879
	50	7	0.70635	0.81947
	50	14	0.72974	0.95829
**KG-1**	2	7	0.30067	0.96438
	2	14	0.5219	0.58321
	2	28	0.80898	0.15018
	10	7	0.35708	0.74383
	10	14	0.39826	1.30951
	10	28	0.80028	0.17432
	50	7	0.44751	0.72441
	50	14	0.63277	0.45384
**Kasumi-1**	2	7	0.07	1.49702
	2	14	0.398	1.39564
	2	28	0.96487	0.52676
	10	7	0.20882	0.87035

	10	14	0.3801	1.45283
	10	28	0.71924	1.52345
	50	7	0.23233	0.91918
	50	14	0.40275	1.4335
**HL-60**	2	7	0.62673	0.50622
	2	14	0.62966	1.24878
	2	28	0.91939	0.32997
	10	7	0.56982	0.65137
	10	14	0.6701	1.03356
	10	28	0.81608	0.90053
	50	7	0.4456	1.10806
	50	14	0.55804	1.71355

A combination index < 1 indicates synergism.

A combination index = 1 indicates an additive effect.

A combination index > 1 indicates antagonism

Table S7. List of cells used

Cells	Histological type	Species	Source[a,b]
Haematological cancer cell lines			
Jurkat	T-cell lymphoid leukaemia	human	ATCC
MOLM-13	acute myelogenous leukaemia	human	DSMZ
KG-1	acute myelogenous leukaemia	human	DSMZ
KG-1a	acute myelogenous leukaemia	human	ATCC
Kasumi-1	acute myeloblastic leukaemia	human	DSMZ
NB4	acute promyelocytic leukaemia	human	ATCC
HL-60	acute promyelocytic leukaemia	human	ATCC
K-562	chronic myelogenous leukaemia	human	ATCC
THP-1	monocytic leukaemia	human	ATCC
PL-21	acute myelogenous leukaemia	human	DSMZ
MV4-11	acute myelogenous leukaemia	human	DSMZ
Solid cancer cell lines			
MDA-MB-231	breast carcinoma	human	BCRJ
MCF-7	breast adenocarcinoma	human	ATCC
4T1	breast carcinoma	mouse	ATCC
HCT116	colorectal carcinoma	human	ATCC
B16-F10	melanoma	mouse	ATCC
A-375	melanoma	human	BCRJ
HepG2	hepatocellular carcinoma	human	ATCC
OVCAR-3	ovarian carcinoma	human	BCRJ

U-87 MG	glioblastoma	human	BCRJ
A549	lung adenocarcinoma	human	BCRJ
PANC-1	pancreas ductal adenocarcinoma	human	BCRJ
DU 145	prostate carcinoma	human	BCRJ
HSC-3	oral squamous cell carcinoma	human	ATCC
CAL 27	oral squamous cell carcinoma	human	ATCC
SSC-4	oral squamous cell carcinoma	human	ATCC
SCC-9	oral squamous cell carcinoma	human	ATCC
SCC-25	oral squamous cell carcinoma	human	ATCC
Noncancer cell lines			
MRC-5	lung fibroblast	human	ATCC
BJ	foreskin fibroblast	human	ATCC
Mutant and its parental cell lines			
BAD KO SV40 MEF	immortalized mouse embryonic fibroblasts with the BAD gene knocked out	mouse	ATCC
WT SV40 MEF	wild-type immortalized embryonic fibroblasts	mouse	ATCC
Primary cells			
PBMC	health peripheral blood mononuclear cells	human	primary cell culture

[a]ATCC denotes the American Type Culture Collection (U.S.A.), DSMZ denotes the Deutsche Sammlung von Mikroorganismen und Zellkulturen (Germany), and BCRJ denotes the Rio de Janeiro Cell Bank (Brazil). [b]Primary cell cultures of

PBMCs were obtained from the peripheral blood of healthy donors via a standard Ficoll density protocol. The PBMCs were subsequently resuspended in RPMI 1640 or DMEM-F12 supplemented with 20% FBS and 1% antibiotics. The cells were plated at 5×10^5 cells/well. Concanavalin A (10 µg/mL, Sigma–Aldrich) was used as a mitogen to trigger cell division in T lymphocytes and was added at the beginning of the culture. The Research Ethics Committee of the Oswaldo Cruz Foundation (Salvador, Bahia, Brazil) approved the protocol (CAAE 16220713.2.0000.0040).

Table S8. Antibodies used for flow cytometry or confocal imaging

Epitope	Fluorochrome	Reactivity	Application	Clone	Catalogue number	Manufacturer
Active caspase-3	FITC	Human/ Mouse	Intracellular staining	C92-605	559341	BD Pharmingen™
CD11b	PE	Human	Cell surface staining	D12	347557	BD™
CD123	BV605	Human	Cell surface staining	7G3	564197	BD Horizon™
CD13	PE-CF594	Human	Cell surface staining	WM15	562491	BD Horizon™
CD33	BV510	Human	Cell surface staining	WM53	563257	BD Horizon™
CD34	PE	Human	Cell surface staining	8G12	348057	BD™
CD38	BV421	Human	Cell surface staining	HIT2	562444	BD Horizon™
CD45 (hCD45)	PE	Human	Cell surface staining	2D1	368509	BioLegend

CD45 (mCD45)	FITC	Mouse	Cell surface staining	30-F11	103107	BioLegend
Cleaved PARP (Asp214)	PE	Human	Intracellular staining	F21-852	552933	BD Pharmingen™
IgG1, κ Isotype Control	PE	-	Cell surface staining	MOPC-21	556650	BD Pharmingen™
NF-κB p65	PE	Human	Intracellular staining	14G10A21	653004	BioLegend
Phospho-NF-κB p65 (Ser529)	AF488	Human	Intracellular staining	K1089512 50	558421	BD Phosflow™
Phospho-NF-κB p65 (Ser536)	FITC	Human	Intracellular staining	NFKBp65S 536-B7	MA5-37157	Thermo Scientific

Table S9. Antibodies used for western blotting

Epitope	Clone	Catalogue number	Manufacturer
Anti-mouse IgG, HRP-linked	-	#7076	Cell Signaling Technology
Anti-rabbit IgG, HRP-linked	-	#7074	Cell Signaling Technology
BCL-2	-	#2872	Cell Signaling Technology
Caspase-3	-	#9662	Cell Signaling Technology
Cleaved caspase-3	-	#9661	Cell Signaling Technology
GAPDH	-	#97166	Cell Signaling Technology
IKKα	3G12	#11930	Cell Signaling Technology
IKKβ	D30C6	#8943	Cell Signaling Technology
IκBα	L35A5	#4814	Cell Signaling Technology
NF-κB p65	D14E12	#8242	Cell Signaling Technology

PARP	-	#9542	Cell Signaling Technology
Phospho-IKKα/β (Ser176/180)	16A6	#2697	Cell Signaling Technology
Phospho-IκBα (Ser32)	14D4	#2859	Cell Signaling Technology
Phospho-NF-κB p65 (Ser536)	93H1	#3033	Cell Signaling Technology
